# How long do pathogens persist and survive in water? A systematic review

**DOI:** 10.3389/fmicb.2025.1654785

**Published:** 2025-10-28

**Authors:** Famous K. Sosah, Alex Odoom, Isaac Anim-Baidoo, Eric S. Donkor

**Affiliations:** Department of Medical Microbiology, University of Ghana Medical School, Accra, Ghana

**Keywords:** waterborne pathogens, persistence, survival, water safety, waterborne disease, systematic review, environmental conditions

## Abstract

**Background:**

Water is an important vehicle for transmitting pathogens that can cause waterborne diseases. Depending on environmental conditions, pathogenic microorganisms present in water can survive and persist for varying durations. A systematic understanding of pathogen survival and persistence under diverse environmental conditions is important for assessing water safety and guiding treatment requirements.

**Methods:**

A systematic search of PubMed, Web of Science, Google Scholar, and Scopus databases was conducted to identify studies published from January 1, 1990, to July 17, 2024. Studies were included and analysed if they provided quantitative data on the survival or persistence of bacteria, viruses, parasites, or fungi in different water sources, under varying environmental conditions such as temperature and pH. The PRISMA guidelines were followed, and the risk of bias in each study was evaluated using the Cochrane ROB2 tool.

**Results:**

Of 2,048 initial records, 58 studies met inclusion criteria. Bacteria were the most studied group, with a mean survival of 28 days and persistence up to 621 days, especially at lower temperatures and in freshwater. *E. coli* O157:H7 and Salmonella spp. were the most studied bacteria, persisting for weeks to months in both freshwater and biofilms. Viruses averaged 22 days of survival but could persist up to 1,095 days; Human Adenovirus and Porcine epidemic diarrhea virus were the most frequently studied viral pathogens, while Human Norovirus survived over 70 days in surface water at 37°C. Viral viability decreased significantly at extreme pH levels. Parasites/protozoans, such as *Cryptosporidium parvum* and *Giardia lamblia* only showed survival duration, averaged 30 days survival, enduring extreme conditions and often benefiting from biofilm association. Fungi, though less studied, are emerging waterborne pathogens, with *Candida auris* surviving up to 30 days in water. Lower temperatures, neutral to slightly alkaline pH, and biofilms significantly enhanced pathogen persistence, even in treated water systems.

**Conclusion:**

Pathogenic microorganisms can survive and persist in diverse water environments for extended periods, posing ongoing risks for waterborne disease transmission and highlighting limitations in current water treatment strategies. Strengthening surveillance and disinfection protocols, prioritizing biofilm management strategies, and predictive modeling to enhance waterborne disease prevention and inform public health policies globally.

## Graphical Abstract

**Figure:**
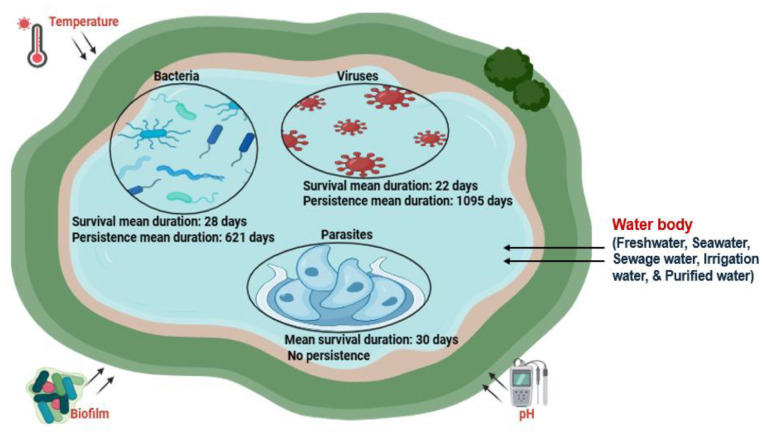
Pathogen survival and persistence in water.

## 1 Introduction

Waterborne diseases are among the most persistent and economically disastrous biological threats to public health (Shayo et al., 2023). Globally, 2.1 billion people lack access to clean and safe drinking water, resulting in 2.2 million deaths from waterborne diseases annually (Shayo et al., 2023). In developing countries, four-fifths of all illnesses are caused by waterborne diseases, with diarrhea being the leading cause of childhood death (Mishra and Sahu, 2022; Shayo et al., 2023). The severity of waterborne diseases is much greater in developing than in developed countries. Owing to limited access to reliable, clean water supplies and proper sanitation, many people drink untreated water from contaminated sources (World Health Organization, 2023; Kunz et al., 2024). Common waterborne pathogens such as *Escherichia coli*, *Salmonella* spp., Norovirus, Hepatitis A, *Giardia lamblia*, and *Cryptosporidium parvum* are responsible for severe gastrointestinal illnesses, which impose a significant economic burden due to healthcare costs, loss of productivity, and reduced quality of life (Collier et al., 2021). The economic burden of these waterborne disease outbreaks is substantial. Waterborne pathogens are estimated to cause an estimated 7.15 million illnesses, 118,000 hospitalizations, and 6,630 deaths in the United States each year, resulting in $3.33 billion in direct healthcare costs (Collier et al., 2021). Biofilm-related pathogens, such as *Legionella* spp., account for 40% of hospitalizations and 50% of deaths, costing $1.39 billion annually in the United States (Collier et al., 2021). These pathogens contaminate water through human activities, animal waste, and agricultural activities, including the use of fertilizers (Collier et al., 2021). Runoff and flooding resulting from expected increases in extreme precipitation, hurricane rainfall, and snowmelt can transport pathogens from these sources into surface waters and groundwater (Nichols et al., 2018; Bagordo et al., 2024). Water contamination from wildlife, such as rodents, birds, deer, and wild pigs, occurs via the feces and urine of infected animals, which are reservoirs of zoonotic pathogens (Kruse et al., 2004). Understanding how long these pathogens can survive in water is essential for developing effective prevention and control measures. Therefore, this study aims to fill that gap by evaluating the duration of pathogen survival in different water environments and assessing how various external conditions, such as temperature and pH, impact pathogen persistence.

## 2 Methods

### 2.1 Search strategy

This systematic review was conducted following the Preferred Reporting Items for Systematic Reviews and Meta-Analysis (PRISMA) guidelines (Page et al., 2021). The PRISMA guidelines offer a detailed checklist and flow diagram that assist in record identification, screening, and evaluation. Between 16th and 17th July 2024, we extensively searched electronic databases, including Scopus, Web of Science, Google Scholar, and PubMed, from January 1, 1990, to July 17, 2024. The rationale for selecting this timeframe was that hardly any relevant studies before 1990 were indexed in databases. A comprehensive search was performed via the following search terms: (“pathogens” OR “parasites” OR “bacteria” OR “viruses” OR “fungi” “protozoan”) AND (“persistence” OR “survival” OR “duration” OR “lifespan”) AND (“wastewater” OR “surface water” OR “bottled water” OR “seawater” OR “river-water” OR “rainwater” OR “municipal water”). Furthermore, the reference lists of the identified articles were examined to identify any additional relevant publications.

### 2.2 Inclusion and exclusion criteria

Based on the search terms, we incorporated studies presenting quantitative data on various pathogens (bacteria, viruses, protozoa, fungi) in different water sources. The types of studies used included primary studies, including experimental, observational, cross-sectional, prospective, and outbreak studies. Studies on the persistence of pathogen strains and pathogens in symbiotic associations with living and non-living organisms in any water body and publications on pathogen persistence in water-containing sediments and water microcosms were also included. The selection of studies was limited to articles that were accessible, available in full text, and published in the English language. There were no geographical limitations. Studies excluded from the review included opinion studies, editorials, letters, commentaries, published review articles, textbooks, and studies that reported only the presence of the pathogen but did not quantitatively provide the duration of persistence, although they were screened for relevant information. Studies on the persistence of pathogen markers were also excluded since these markers are not pathogens themselves. Publications on pathogens persisting in water but affecting fishes and plants with no evidence of being infectious to humans were also excluded.

### 2.3 Study selection

A two-stage screening process was employed to identify relevant studies for inclusion in the review. Firstly, duplicates and irrelevant articles were removed from the identified studies’ titles and abstracts. The remaining studies’ full texts were then carefully reviewed, considering predetermined inclusion and exclusion criteria, in order to ascertain their eligibility. The review included studies that provided original data on pathogen survival or persistence in food, and the findings were extracted. Similarly, studies that detected only the presence of pathogens without estimating the duration of their survival or persistence were excluded, although they were screened for relevant information. This review defines survival as a pathogen’s ability to remain alive and viable for a specific period (up to a year), while persistence is the repeated isolation of a pathogen over a year, maintaining the same molecular or genetic characteristics. Biofilm formation is a crucial factor in providing a stable environment for pathogen survival and persistence. The screening process involved two reviewers, F.K.S. and A.O., adhering to predefined criteria, with a third reviewer consulted to resolve disagreements. This third reviewer ensured consistency, provided an unbiased perspective, and facilitated informed decisions in complex cases. The references of the included articles were then downloaded using the Zotero reference management tool (Corporation for Digital Scholarship, 2022), and the Rayyan tool (Ouzzani et al., 2016) was used to identify duplicate records to facilitate the review process.

### 2.4 Data extraction and analysis

Microsoft Excel 365 software was used to manage the data from the studies reviewed. The data were independently extracted from individual studies via the data abstraction format prepared in Microsoft Excel 365. The extracted information included the author(s), the pathogens studied, the water type or source, the duration of pathogen survival or persistence, the method of detection, and the external conditions influencing the persistence of the pathogen in water. The extracted informations are shown in Supplementary Tables 1-3. Tables were used to visualize the distribution of the study characteristics and findings.

### 2.5 Quality assessment

The risk of bias in each study was evaluated via the Cochrane ROB2 tool (Sterne et al., 2019), and the results were visually presented via the Robvis tool (McGuinness and Higgins, 2020). This quality assessment tool examines five key areas of bias: randomization, deviations from planned interventions, missing outcome data, outcome measurement, and selection of reported results. Assignments of low, high, or some risk of bias to each domain and any discrepancies were resolved through consultation with a second reviewer. A study was considered to have a low risk of bias if all domains received a low-risk classification, high risk if at least one domain was rated as high risk, and some concerns if issues were identified in one or more domains.

## 3 Results

### 3.1 Search results

**FIGURE 1:**
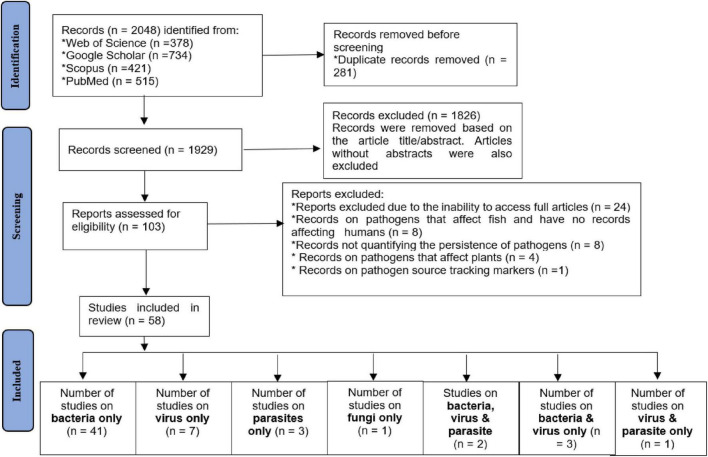
PRISMA flow diagram for the identification, screening, and evaluation of the articles included in the study.

The initial search of the online databases identified a total of 2048 publications from various databases, including Web of Science (*n* = 378), Google Scholar (*n* = 734), Scopus (*n* = 421), and PubMed (*n* = 515). After removing duplicates (*n* = 281), the titles and abstracts of the remaining 1929 records were screened. Among these, 1826 articles were excluded because they did not meet the established inclusion criteria. A total of 103 full-text articles were subsequently assessed for eligibility, of which 58 met the inclusion criteria for the review (Figure 1). These 58 articles included 41 studies focused solely on bacteria; 7 studies focused solely on viruses; 3 studies focused solely on parasites; 1 study focused solely on fungi; 2 studies focused only on bacteria, viruses, and parasites; 3 studies focused only on bacteria and viruses; and 1 study focused only on viruses and parasites (Figure 1).

### 3.2 Survival and persistence of bacteria in freshwater systems

Table 1 shows the various bacterial pathogens, their persistence and survival in freshwater systems and the environmental conditions influencing their persistence and survival. Of the 58 studies included in this review, 46 investigated bacterial persistence and survival across water sources; of these, 19 (41.3%) focused on treated and untreated freshwater.

**TABLE 1:** Survival and persistence of pathogens in freshwater systems.

Pathogen	Type	Water source	Duration	Condition	Reference
*Arcobacter butzleri*	Bacteria	Sterile and non-sterile river water	28 days	(15 and 25)°C	Banihashemi et al., 2017
Avian influenza virus (LPAIV H1N1)	Virus	Artificial stream	14 days	10 °C–13 °C	Perlas et al., 2023
*Campylobacter jejuni*	Bacteria	Sterile and non-sterile river water	28 days	15 °C	Banihashemi et al., 2017
*Campylobacter* spp.	Bacteria	Lake water microcosm	28 days	15 °C, 25°C	Ahmed et al., 2021
*Colpoda* spp.	Parasite	Chlorinated water	6 months	3 °C–21 °C	Baré et al., 2011
*Cryptosporidium parvum*	Parasite	Drinking water biofilm	34 days	10 °C, pH 7.67	Helmi et al., 2008
*Cryptosporidium parvum*	Parasite	Drinking water without biofilm	7 days	10 °C, pH 7.67	Helmi et al., 2008
*Cryptosporidium parvum* (oocyst)	Parasite	Filtered and unfiltered water	21 days	15 °C, 25 °C	Ahmed et al., 2021
*E. coli*	Bacteria	Freshwater sediment	90 days	–	Haller et al., 2009
*E. coli*	Bacteria	Lake water microcosm	28 days	15 °C, 25 °C	Ahmed et al., 2021
*E. coli*	Bacteria	Stream water	25 days	18 °C	Haack et al., 2015
*E. coli*	Bacteria	River water	11 days (culture); > 42 days (qPCR)	20 ± 2 °C pH 7.4–8.0	Martín-Díaz et al., 2017
*E. coli*	Bacteria	River water	37 days	8 °C, 18 °C	Essert et al., 2023
*E. coli O157:H7*	Bacteria	Non-sterile lake and river microcosm	60 days	10 °C	Avery et al., 2008
*E. coli O157:H7*	Bacteria	Sterilized river water	28 days	5 °C, 25 °C and 37 °C	Kabir et al., 2020
*E. coli O157:H7*	Bacteria	Untreated river water	109 days	37 °C, pH 2.5–3.0	Scott et al., 2006
*E.coli*	Bacteria	Stormwater water	15 days	37 °C (wildfire residues)	Valenca et al., 2020
*E.coli*	Bacteria	Storm water	>15 days	37 °C (unburned soil particles	Valenca et al., 2020
*E.coli O157:H7*	Bacteria	Sterilized puddle water microcosm	64 days	10 °C	Avery et al., 2008
*E.coli O157:H7*	Bacteria	Non-sterilized puddle water microcosm	At least 18 days	10 °C	Avery et al., 2008
*Enterococcus* spp.	Bacteria	Freshwater sediment	90 days	–	Haller et al., 2009
*Francisella tularensis* subsp. *holarctica* LVS strain	Bacteria	Sterile tap water	21 days	8 °C	Rice, 2015
*Francisella tularensis* subsp. *holarctica NY98*	Bacteria	Pond water	31 days	–	Rice, 2015
*Francisella tularensis* subsp. *holarctica NY98*	Bacteria	Sterile tap water	28 days	8 °C	Rice, 2015
*Francisella tularensis* subsp. *holarctica NY98*	Bacteria	Filter-sterilized brook water	At most 10 days	4 °C	Rice, 2015
*Giardia lamblia*	Parasite	Treated wastewater biofilm and drinking water	>34 days	–	Helmi et al., 2008
*Helicobacter pylori*	Bacteria	Drinking water biofilm	31 days	15 and 20 °C	Gião et al., 2008
Human adenovirus	Virus	Ground water	>3 years	–	Anderson et al., 2011
Human adenovirus (40/41)	Virus	Unfiltered lake water microcosm	28 days	15 °C, 25 °C	Ahmed et al., 2021
Human Norovirus	Virus	Surface water	>70 days	37 °C	Anderson et al., 2011
Human Norovirus	Virus	Ground water	>3 years	–	Anderson et al., 2011
*Klebsiella oxytoca*	Bacteria	River water	37 days	8 °C	Essert et al., 2023
*Klebsiella pneumoniae* (hypermucoviscous)	Bacteria	Freshwater microcosms	44 days	10 °C and 20 °C	Soto et al., 2020
*Klebsiella pneumoniae* (non-hypermucoviscous)	Bacteria	Freshwater microcosms	14 days	10 °C and 20 °C	Soto et al., 2020
*Legionella pneumophila*	Bacteria	Drinking water	850 days	22 ± 1°C	Shaheen et al., 2019
*Leptospira interrogans* serovar	Bacteria	Spring water	28 days	29 °C	Casanovas-Massana et al., 2018
*Listeria monocytogenes*	Bacteria	River water	28 days	5 °C, 25 °C, 37 °C	Kabir et al., 2020
*Listeria monocytogenes*	Bacteria	Drinking water and biofilm	35–47 days	10 °C	Bjergbæk et al., 2021
*Listeria monocytogenes*	Bacteria	Drinking water	At least 28 days	10 °C	Bjergbæk et al., 2021
*Mycobacteria avium* (non-tuberculosis mycobacteria)	Bacteria	Drinking water	26 months	9.9 °C–21.5 °C	Hilborn et al., 2006
Non-HMV *Klebsiella pneumoniae*	Bacteria	Freshwater microcosms	14 days	20 °C	Soto et al., 2020
Non-typhoidal *Salmonella enterica* serovars	Bacteria	River water	28 days	5 °C, 25 °C, 37 °C	Kabir et al., 2020
*Salmonella enterica*	Bacteria	Sterile and non-sterile River water	28 days	15 °C	Banihashemi et al., 2017
*Salmonella* spp.	Bacteria	Spring lake water	23 days	–	Gaertner et al., 2011
*Salmonella typhimurium*	Bacteria	Microcosm with stream water	>164 h	40 °C (sediment particle attached)	Wang et al., 2018
*Salmonella typhimurium*	Bacteria	Microcosm with stream water	>120 h	40 °C (free floating)	Wang et al., 2018
*Salmonella typhimurium*	Bacteria	Microcosm with stream water	5 h	50 °C (free floating)	Wang et al., 2018
*Salmonella typhimurium*	Bacteria	Microcosm with stream water	>21 h	50 °C (sediment particle attached)	Wang et al., 2018
*Salmonella typhimurium*	Bacteria	Microcosm with stream water	<1.5 h	60 °C (free floating)	Wang et al., 2018
*Salmonella typhimurium*	Bacteria	Microcosm with stream water	2 h	60 °C (sediment particle attached)	Wang et al., 2018
SARS-CoV	Virus	Dechlorinated tap water	2 days	20 °C	Basavaraju et al., 2021
SARS-CoV	Virus	Dechlorinated tap water	14 days	4 °C	Basavaraju et al., 2021
*Staphylococcus aureus*	Bacteria	River water	7 days	8 °C	Essert et al., 2023
*Staphylococcus aureus*	Bacteria	River water	3 days	18 °C	Essert et al., 2023
*Trichobilharzia szidati cercariae*	Parasite	Dechlorinated tap water	204 h	5 °C	Al-Jubury et al., 2020
*Trichobilharzia szidati cercariae*	Parasite	Dechlorinated tap water	108 h	10 °C	Al-Jubury et al., 2020
*Trichobilharzia szidati cercariae*	Parasite	Dechlorinated tap water	96 h	15 °C	Al-Jubury et al., 2020
*Trichobilharzia szidati cercariae*	Parasite	Dechlorinated tap water	60 h	20 °C	Al-Jubury et al., 2020
*Trichobilharzia szidati cercariae*	Parasite	Dechlorinated tap water	48 h	25 °C	Al-Jubury et al., 2020
*Trichobilharzia szidati cercariae*	Parasite	Dechlorinated tap water	36 h	30 °C	Al-Jubury et al., 2020
Vaccinal poliovirus type 1 (infectious)	Virus	Drinking water biofilm	6 days	10 °C	Helmi et al., 2008
Vancomycin-resistant enterococci faecium strain	Bacteria	Filtered river water	7 days	–	Young et al., 2019
Virulent *Aeromonas hydrophila* (vAh)	Virus	Pond water sediment	28 days	28 °C	Tuttle et al., 2023
*Yersinia enterocolitica*	Bacteria	Sterile and non-sterile river water	28 days	15 °C	Banihashemi et al., 2017
*Burkholderia cenocepacia*	Bacteria	Atlantic Ocean	1 month	20 °C	Shteinberg et al., 2015

Freshwater systems, including rivers, lakes, streams, and drinking water supplies, serve as reservoirs for indicator microorganisms and pathogens beyond the expected short durations. *Escherichia coli*, a widely used indicator of fecal contamination, has demonstrated resilience for up to 28 days in both sterile and non-sterile lake microcosms at 15 °C–25 °C (Ahmed et al., 2021), and survives up to 90 days when associated with sediments (Haller et al., 2009). A similar sediment-enhanced endurance was observed for *Enterococcus* species, which also survived 90 days (Haller et al., 2009) under comparable conditions, but in sediment free freshwater, it survived at most 5 days at 25 °C (Mahler et al., 2000). This thus explains the role of sediment as a protective niche that prolongs bacterial viability. Temperature has an effect on bacterial survival in freshwater systems. *E. coli* in rivers endured 37 days at 8 °C versus 25 days at 18 °C (Banihashemi et al., 2017), and this could highlight that cooler waters retard the die-off rate of pathogens. *Campylobacter jejuni* exhibited parallel resilience, surviving 28 days in river and lake waters held at 15 °C–25 °C. *Salmonella enterica* strains survived for 28 days across a broad thermal range (5 °C–37 °C) in river water, but only 2 days in marine water at 22 °C and 27 °C (Nayak et al., 2015) with sediment-attached cells outlasting planktonic counterparts (Banihashemi et al., 2017; Ahmed et al., 2021). *Listeria monocytogenes* likewise survived for nearly a month in river water and extended to 35–47 days within drinking water biofilms at 10 °C (Banihashemi et al., 2017), and this may be due to the protective niche of biofilms that shields pathogens from environmental stressors. These patterns highlight how freshwater sediments and biofilms serve as critical reservoirs for enteric bacteria, while lower temperatures uniformly enhance their persistence. Such extended survival has important implications for water quality monitoring and public health risk assessments.

#### 3.2.1 Frequency of bacteria in freshwater systems

**FIGURE 2:**
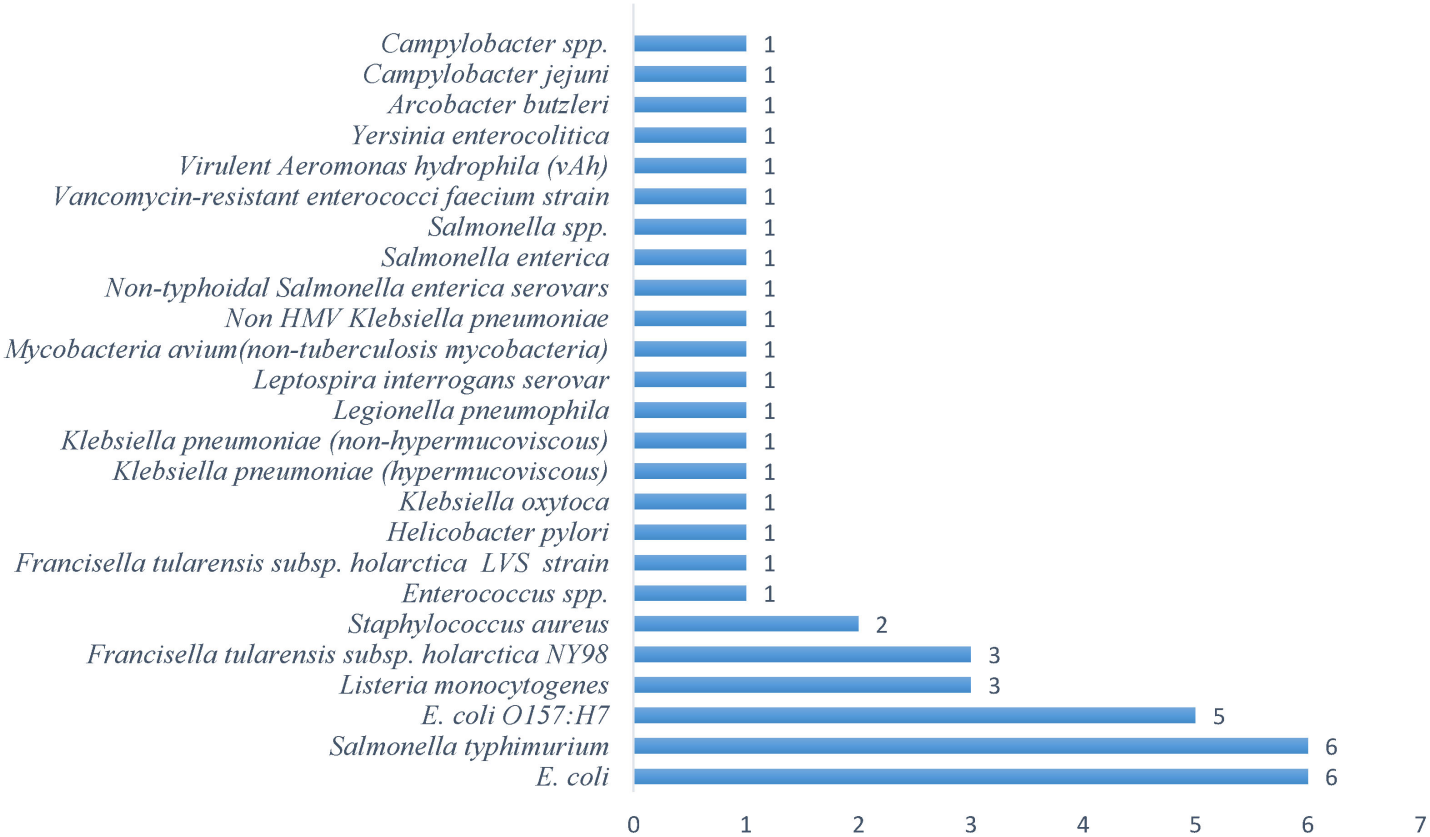
Frequency of bacterial pathogens in freshwater systems.

*E. coli O157:H7* is the third most studied pathogen, with 5 occurrences (Figure 2), warranting its attention due to its production of shiga toxins that can cause life-threatening complications in both recreational and drinking water contexts. This research emphasis demonstrates the scientific community’s focus on pathogens with the greatest potential for mass casualty events. A distinct secondary tier comprises opportunistic pathogens that pose particular threats to vulnerable populations. *Listeria monocytogenes* and *Francisella tularensis* subsp. *Holarctica NY98* (3 occurrences), while *Staphylococcus aureus* contributes 2 occurrences. This pattern reveals growing recognition that freshwater safety extends beyond healthy individuals to encompass immunocompromised populations who face disproportionate risks from organisms that might otherwise be considered benign. *Campylobacter* species, *Yersinia enterocolitica*, *Legionella pneumophila*, and *Mycobacteria avium* each received minimal attention with single occurrences. This distribution likely reflects their sporadic involvement in waterborne outbreaks rather than their potential severity, as these pathogens can cause serious infections under specific environmental conditions or in vulnerable individuals. The frequency distribution of pathogens (Figure 2) with the concentration of studies on *E.coli* variants and *Salmonella species* shows their dual importance as both significant pathogens and reliable indicators of fecal contamination, making them essential targets for water quality monitoring.

### 3.3 Survival and persistence of viral pathogens in freshwater systems

The survival and persistence of viruses in freshwater sources were reported in 13 studies, of which 7 (53.85%) focused on viral survival in the treated and untreated freshwater (Table 1). Temperature conditions influence viral survival, with low-pathogenic Avian influenza (LPAIV) H1N1 remaining infectious for 14 days in an artificial stream at 10 °C–13 °C (Perlas et al., 2023), and SARS-CoV survived 14 days at 4 °C in dechlorinated tap water compared to just 2 days at 20 °C (Basavaraju et al., 2021). Conversely, Human Norovirus exhibited endurance, surviving beyond 70 days in surface water at 37 °C (Anderson et al., 2011) and mirroring this multi-year stability in groundwater alongside Human Adenovirus, which also survived over 3 years in subsurface aquifers (Anderson et al., 2011). In unfiltered lake microcosms, Adenovirus serotypes 40/41 retained infectivity for 28 days at both 15 °C and 25 °C (Ahmed et al., 2021), reflecting the robustness of non-enveloped viruses in freshwater systems. Protective matrices further extend virus longevity; Poliovirus type 1 survived 6 days within drinking water biofilms at 10 °C (Helmi et al., 2008), while an *Aeromonas hydrophila* phage endured 28 days in pond sediment at 28 °C (Tuttle et al., 2023), illustrating sediment association as a critical niche for viral survival in freshwater systems. These patterns reveal that groundwater, biofilms, and sediments serve as enduring viral reservoirs, challenging conventional water treatment strategies.

#### 3.3.1 Frequency of viral pathogens in freshwater systems

**FIGURE 3:**
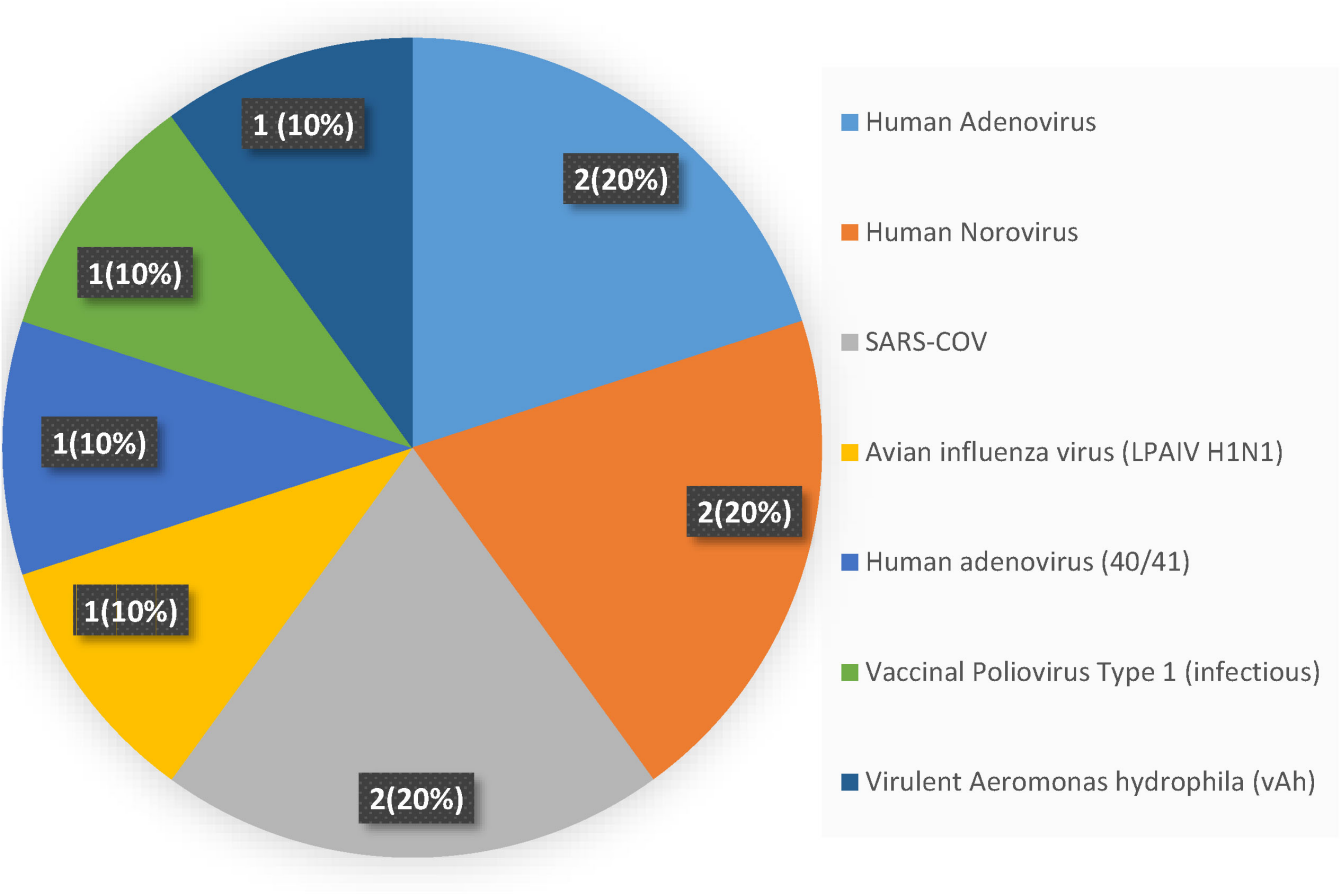
Frequency of viral pathogens in the freshwater system.

Viral surveillance in freshwater environments showed a hierarchy among pathogenic species. Three viruses, Human Adenovirus, Human Norovirus, and SARS-CoV, collectively accounted for 60% of all positive detections, with each contributing equally at 20% of total occurrences (Figure 3). This concentration could suggest these pathogens represent the primary viral threats in freshwater systems, likely reflecting their environmental stability, widespread human shedding, and resistance to standard water treatment processes. The remaining viral diversity is distributed evenly among four less prevalent species: Avian Influenza H1N1, Human Adenovirus serotypes 40/41, Vaccinal Poliovirus type 1, and virulent *Aeromonas hydrophila* phage, each comprising 10% of detections. This uniform distribution among secondary pathogens contrasts sharply with the concentrated prevalence of the dominant trio, creating a distinct two-tier pattern in freshwater viral ecology. The predominance of Human Adenovirus and Norovirus aligns with their known characteristics as persistent enteric viruses that resist environmental degradation and maintain infectivity across varied aquatic conditions (Jiang, 2006; Seitz Scot et al., 2011). SARS-CoV’s inclusion among the most detected viruses reflects both its environmental persistence and the intensive surveillance efforts during the COVID-19 pandemic, which may have enhanced detection sensitivity for this respiratory pathogen in water systems.

### 3.4 Survival and persistence of parasite (protozoans) in freshwater systems

Analysis of 6 studies shows that protozoan and helminth parasites can endure in freshwater far beyond conventional expectations, often aided by biofilms and cooler temperatures. The free-living protozoan *Colpoda* spp. remained viable for 6 months in chlorinated water across a 3 °C–2 °C range (Baré et al., 2011). Embedded within treated wastewater biofilms and drinking water systems, *Giardia lamblia* cysts survived for over 34 days, while *Cryptosporidium parvum* oocysts survived 34 days when associated with biofilms but lost infectivity after just 1 week in biofilm-free water under identical conditions, a stark demonstration of biofilm protection (Helmi et al., 2008). Even without biofilms, *Cryptosporidium parvum* withstood 21 days in both filtered and unfiltered lake microcosms at 15 °C–25 °C (Ahmed et al., 2021; Figure 3), illustrating resilience to temperature fluctuations and filtration processes. Larval *Trichobilharzia szidati* exhibited a temperature-dependent decline, surviving up to 204 h at 5 °C but only 36 h at 30 °C in dechlorinated tap water (Al-Jubury et al., 2020). These patterns emphasize that biofilm matrices and lower temperatures substantially extend parasite survival, challenging water treatment strategies to target these refuges to ensure effective pathogen removal.

#### 3.4.1 Frequency of parasites in freshwater systems

**FIGURE 4:**
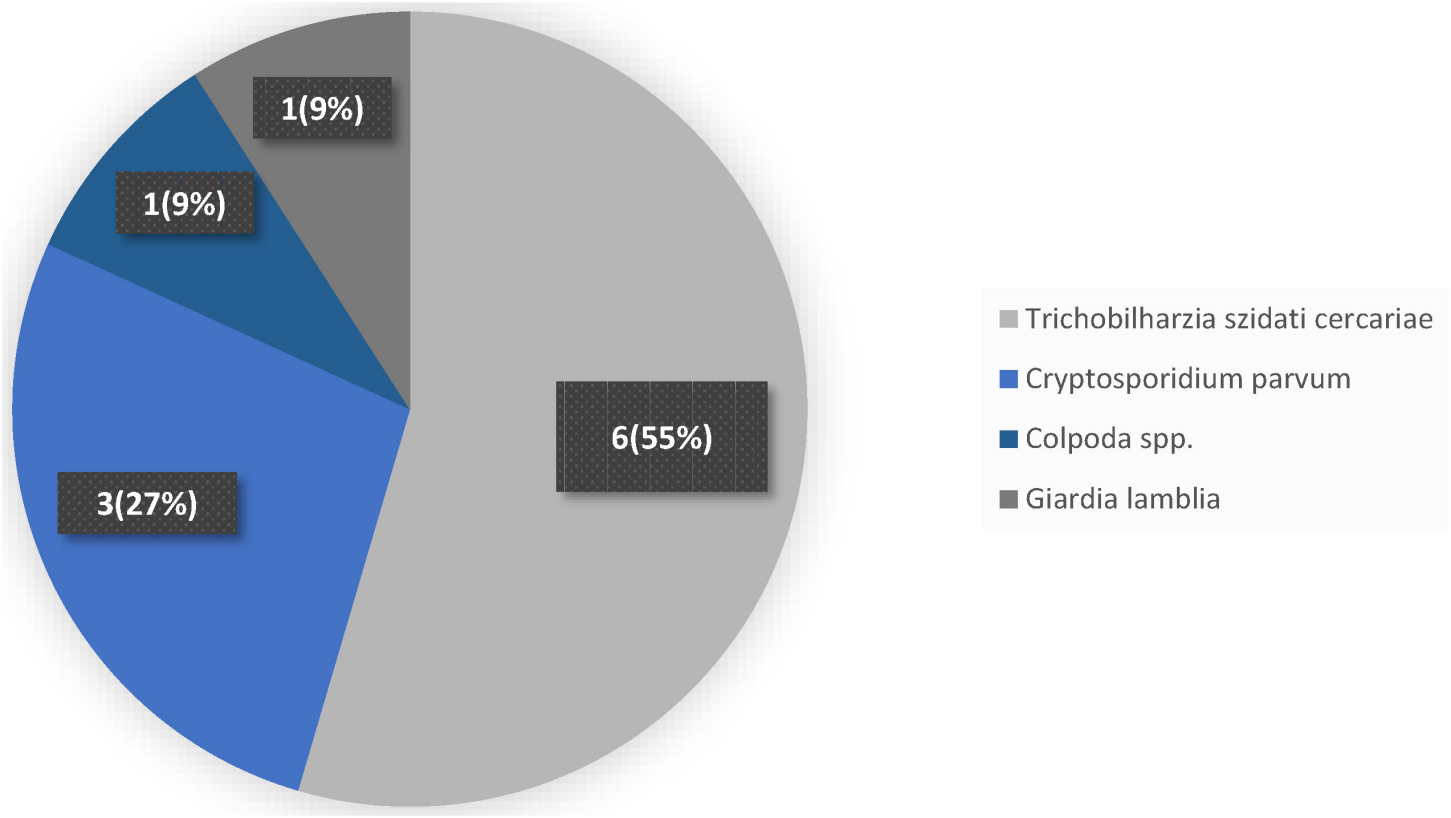
Frequency of parasites in freshwater system.

In Figure 4, parasite pathogens in the freshwater system showed a dominance by *Trichobilharzia szidati* cercariae, which accounted for more than half of all detections. Out of 11 total incidences recorded, 6 were identified as *Trichobilharzia szidati* cercariae, representing 55% of the parasite load. This prevalence of avian schistosome larvae in these waters raises concerns about potential swimmer’s itch outbreaks among recreational users. Following *Trichobilharzia szidati* cercariae, *Cryptosporidium parvum* emerged as the second most frequent parasite, with 3 detections comprising 27% of the total. Although less common than the schistosome cercariae, the presence of *Cryptosporidium parvum* is nonetheless significant given its well-known capacity to cause gastrointestinal illness in both humans and livestock. In contrast, two protozoan genera appeared only sporadically. *Colpoda* spp. and *Giardia lamblia* were each detected once, together making up the remaining 18% of parasite occurrences. The single *Colpoda* spp. detection (9%) highlights the occasional presence of free-living ciliates in the water column, while the solitary *Giardia lamblia* finding (also 9%) could signal a potential public health risk due to this well-established waterborne pathogen.

### 3.5 Survival and persistence of bacteria in marine water systems

Eight (17.4%) studies focus on marine water environments, supporting the persistence of diverse bacterial pathogens for periods ranging from hours to several months, with survival strongly influenced by both species characteristics and ambient conditions (Table 2).

**TABLE 2:** Survival and persistence of pathogens in marine (seawater) systems.

Pathogen	Type	Water source	Duration	Condition	Reference
*Burkholderia cenocepacia*	Bacteria	Dead sea	24 h	20 °C	Shteinberg et al., 2015
*E. coli*	Bacteria	Sea water	7 days	20 ± 2 °C, pH 8.0 ± 0.1.	Ballesté et al., 2024
*E. coli*	Bacteria	Seawater	5 days	10 ± 0.1 °C	Williams et al., 2007
*Francisella tularensis (*subsp*.halarctica* and subsp. *Tularensis)*	Bacteria	Saline solution	24 weeks	4 °C	Golovliov et al., 2021
*Francisella tularensis (*subsp*.halarctica* and subsp. *Tularensis)*	Bacteria	Saline solution	14 weeks	20 °C	Golovliov et al., 2021
Non-HMV *Klebsiella pneumoniae*	Bacteria	Marine water	7 days	20 °C	Soto et al., 2020
*Klebsiella pneumoniae* (hypermucoviscous and non-hypermucoviscous)	Bacteria	Sea water	7 days	10 °C and 20 °C	Soto et al., 2020
*Pseudomonas aeruginosa*	Bacteria	Atlantic Ocean	1 month	20 °C	Shteinberg et al., 2015
*Pseudomonas aeruginosa*	Bacteria	Dead sea	24 h	20 °C	Shteinberg et al., 2015
*Salmonella enterica* serovar *typhimurium*	Bacteria	Filtered seawater	74 weeks	12 °C	Davidson et al., 2015
*Salmonella* sp.	Bacteria	Tidal creek	3 months	4.46 °C–38.56 °C	Jones et al., 2018
*Salmonella typhimurium*	Bacteria	Unfiltered seawater	32 weeks	12 °C	Davidson et al., 2015
*Listeria monocytogenes*	Bacteria	Seaweed-associated water	7 days	4 °C, 10 °C	Akomea-Frempong et al., 2023
*Listeria monocytogenes*	Bacteria	Seaweed-associated water	8 h	22°C	Akomea-Frempong et al., 2023
Shigatoxigenic *E.coli* (STEC)	Bacteria	Seaweed-associated water	7 days	4 °C, 10 °C	Akomea-Frempong et al., 2023
Shigatoxigenic *E.coli* (STEC)	Bacteria	Seaweed-associated water	8 h	22 °C	Akomea-Frempong et al., 2023
*Vibrio* spp.	Bacteria	Seaweed-associated water	7 days	4 °C, 10 °C	Akomea-Frempong et al., 2023

In the Atlantic Ocean at 20 °C, *Burkholderia cenocepacia* and *Pseudomonas aeruginosa* remained culturable for up to 1 month, whereas in the hypersaline Dead Sea under the same temperature, both species declined to undetectable levels within 24 h, marked by the impact of salinity extremes on viability (Shteinberg et al., 2015). Gram-negative enteric bacteria exhibit variable persistence depending on temperature and salinity. *Escherichia coli* survived up to 7 days in seawater at 2 °C (pH 8.0), but only 5 days at 10 °C, indicating paradoxically extended persistence at lower temperatures that slow metabolic activity and inactivation processes (Ballesté et al., 2024). Similarly, *Klebsiella pneumoniae*, including hypermucoviscous and non-hypermucoviscous strains, endured for 7 days in marine water at both 10 °C and 20 °C (Soto et al., 2020), and this could suggest that mucoid phenotypes confer no significant advantage under these conditions. Certain zoonotic and human-pathogenic species demonstrated prolonged survival in low-nutrient saline media. The infectious *Francisella tularensis* subsp. *tularensis* persisted in saline solution for 24 weeks at 4 °C and for 14 weeks at 20 °C, which may indicate the risk of long-term environmental reservoirs in cold marine-influenced waters (Golovliov et al., 2021). *Salmonella enterica* serovar *typhimurium* similarly exhibited remarkable resilience, surviving 74 weeks in filtered seawater at 12 °C and 32 weeks in unfiltered seawater at the same temperature, while other *Salmonella* spp. persisted for 3 months in tidal creek systems, 12 months in sea water microcosms at pH of 8 (Chakroun et al., 2017) and fluctuating between 4.46 °C and 38.56 °C (Davidson et al., 2015; Jones et al., 2018).

In association with macroalgae, pathogens such as *Listeria monocytogenes*, Shigatoxigenic *E. coli*, and *Vibrio* spp. displayed dual-phase survival patterns: enduring for 7 days at lower temperatures (4 °C and 10 °C) but declining to undetectable levels within 8 h and 72 hours at 22 °C (Richards et al., 2012; Akomea-Frempong et al., 2023; Table 2). Likewise in artifical seawater, *Vibrio parahaemolyticus* survived at longer (21 days) at lower temperature (6 °C) but 12 days at 8 °C (Vasudevan and Venkitanarayanan, 2006). This suggests that biofilm formation on seaweed surfaces may protect cells under cool conditions, yet higher temperatures accelerate inactivation.

#### 3.5.1 Frequency of bacteria in marine water systems

**FIGURE 5:**
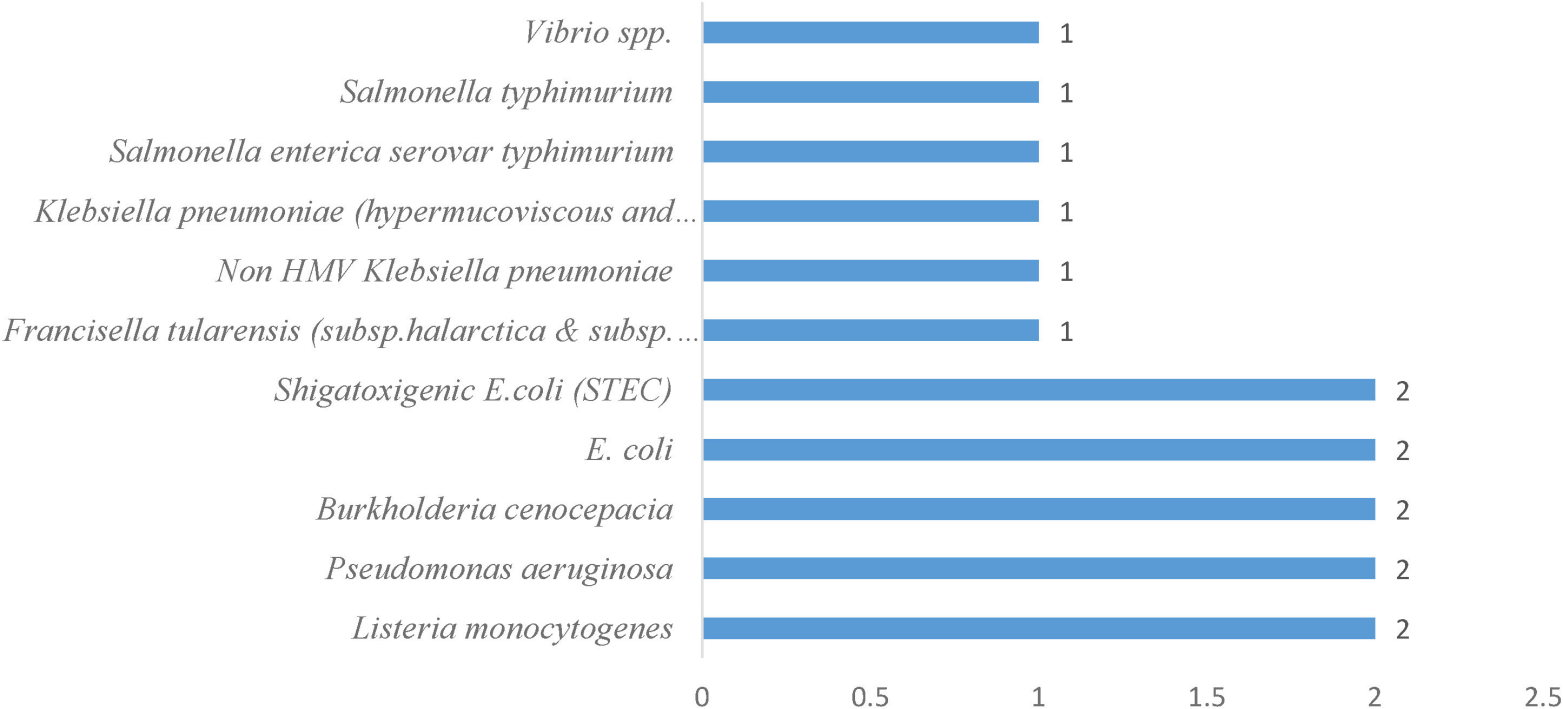
Frequency of bacterial pathogens in the marine water system.

Among the pathogens identified in the marine water systems, *Listeria monocytogenes, Pseudomonas aeruginosa, Burkholderia cenocepacia, Escherichia coli*, and Shiga toxin-producing *E. coli* (STEC) each have 2 occurrences in marine environments (Figure 5). In contrast, *Vibrio* spp., *Salmonella typhimurium*, *Salmonella enterica* serovar *typhimurium*, both hypermucoviscous and non-hypermucoviscous strains of *Klebsiella pneumoniae*, non-HMV *Klebsiella* pneumoniae, and *Francisella tularensis* (including subspecies halarctica and subspecies tularensis) with one occurrence, indicating less frequent but still notable occurrences. The prominence of *Listeria monocytogenes* and *Pseudomonas aeruginosa* in particular aligns with their known adaptability to aquatic habitats and ability to persist under variable salinity and temperature conditions (Metcalf et al., 2023). Likewise, the detection of *Burkholderia cenocepacia and* both forms of *E. coli*, typical indicators of fecal contamination, reflects the influence of terrestrial runoff and anthropogenic inputs on marine water quality. The presence of STEC, which poses significant public health risks through the production of Shiga toxins, further emphasizes the importance of continuous monitoring, as its repeated isolation suggests potential reservoirs or recurring sources of contamination.

In comparison, organisms such as *Vibrio* spp. and *Francisella tularensis* are traditionally associated with specific ecological niches and hosts; their solitary detection may correspond to transient blooms or isolated contamination events. Similarly, the singular recoveries of multiple *Salmonella* spp. strains and various *Klebsiella pneumoniae* phenotypes point to episodic influxes rather than endemic populations. Taken together, these frequency patterns outline a dual concern: the persistent presence of opportunistic and enteric bacteria in marine waters, alongside sporadic incursions of other pathogens whose ecological and epidemiological significance warrants further investigation.

### 3.6 Survival, persistence and frequency of pathogens in sewage water systems

In untreated sewage, *Escherichia coli* and Enterococci each retained viability for 28 days at both 25 °C and 35 °C, in raw wastewater environments (McQuaig et al., 2009; Table 3). The gene of Mycobacterium ulcerans persisted above 27 months in water detritus at 8 °C (Bratschi et al., 2014; Supplementary Table 1).

**TABLE 3:** Survival and persistence of pathogens in sewage water systems.

Pathogen	Type	Water source	Duration	Condition	Reference
*E.coli*	Bacteria	Sewage water	28 days	25 and 35 °C	McQuaig et al., 2009
*Enterococci*	Bacteria	Sewage water	28 days	25 and 35 °C	McQuaig et al., 2009
*Leptospira interrogans* serovar	Bacteria	Sewage water	8 days	29 °C	Casanovas-Massana et al., 2018
*Serratia marcescens*	Bacteria	Sink drain water	1–4 weeks	–	Bourdin et al., 2024
*Stenotrophomonas maltophilia*	Bacteria	Sink drain water	3 weeks	–	Bourdin et al., 2024
Human adenovirus	Virus	Sewage water	28 days	25 and 35 °C	McQuaig et al., 2009
Human polyomaviruses	Virus	Sewage water	28 days	25 and 35 °C	McQuaig et al., 2009
*Acinetobacter baumannii*	Bacteria	Sterilized effluent wastewater	50 days	–	Hrenovic et al., 2016
SARS-CoV	Virus	Wastewater	2 days	20 °C	Basavaraju et al., 2021
SARS-CoV	Virus	Wastewater	14 days	4 °C	Basavaraju et al., 2021

**FIGURE 6:**
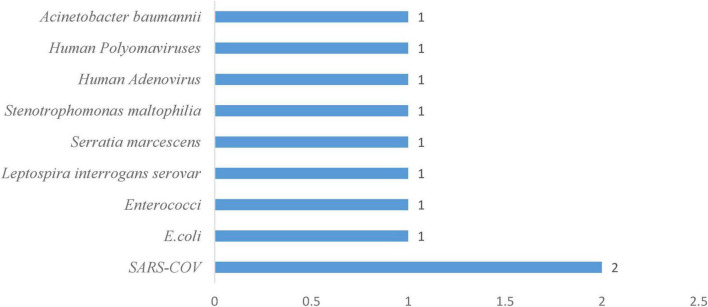
Frequency of pathogens in sewage water system.

Similarly, *Leptospira interrogans* serovars, a pathogenic spirochete, survived for 8 days at 29 °C (Casanovas-Massana et al., 2018). Across multiple sewage samples, SARS-CoV emerged as the most frequently detected pathogen, with 2 observations (Figure 6). Its presence in wastewater may be a potential early indicator of community-level coronavirus transmission. All other organisms, *E. coli*, Enterococci, *Leptospira interrogans* serovars, *Serratia marcescens*, *Stenotrophomonas maltophilia, Acinetobacter baumannii*, Human adenovirus, and Human polyomaviruses, were each recorded once (Figure 6), reflecting their sporadic yet significant presence in sewage water. Domestic plumbing systems also serve as long-term reservoirs for opportunistic bacteria. In sink drain water, *Serratia marcescens* remained recoverable for 1–4 weeks (Bourdin et al., 2024), Other viral pathogens exhibited comparable resilience: Human adenoviruses and polyomaviruses maintained infectivity for 28 days at both 25 °C and 35 °C (McQuaig et al., 2009), which presupposes that typical sewage temperatures do not readily inactivate these viruses. Moreover, cold temperature seems to prolong coronavirus survival in wastewater. SARS-CoV showed a strong temperature dependency; its infectivity declined after 2 days at 20 °C but persisted for up to 14 days at 4 °C. Even following mechanical and chemical treatment, certain bacteria persist. *Acinetobacter baumannii* survived in sterilized effluent for 50 days (Hrenovic et al., 2016).

### 3.7 Duration and frequency of pathogens in irrigation water systems

**FIGURE 7:**
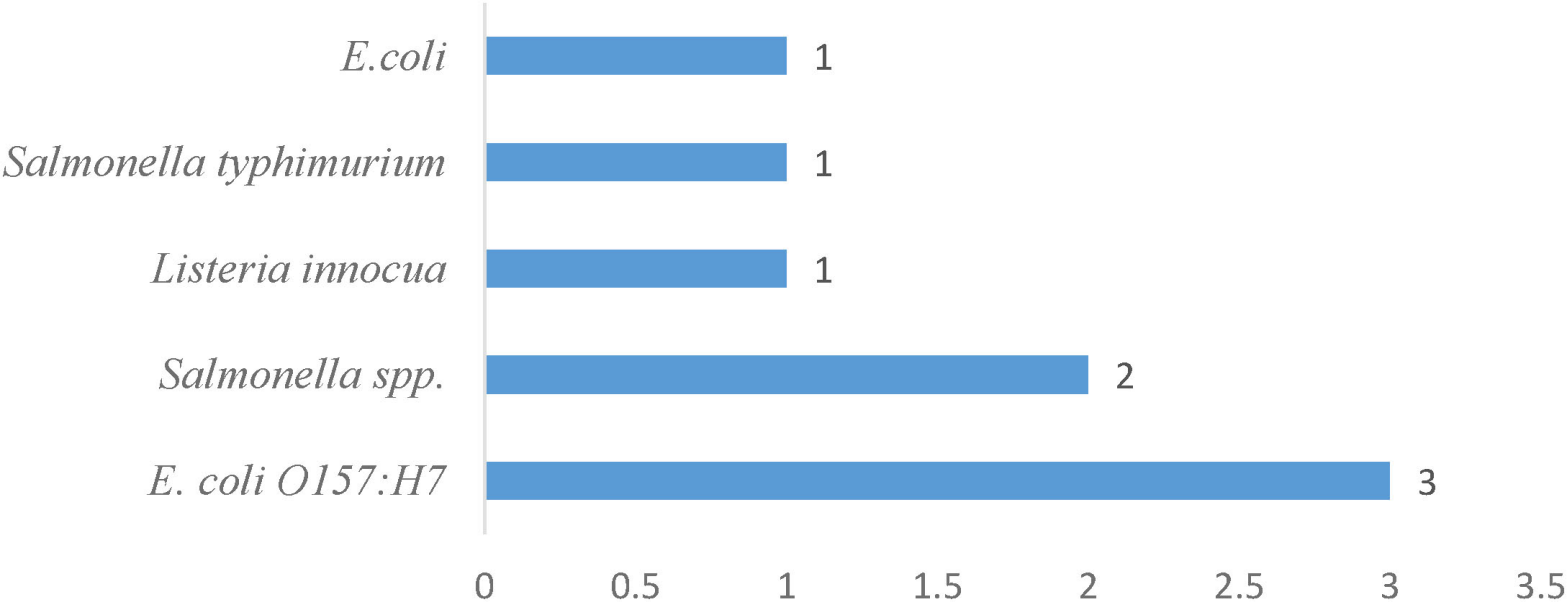
Frequency of pathogens in the irrigation water system.

In Table 4, the survival of 5 bacterial pathogens in irrigation water was assessed under various treatment and storage conditions. Generic *Escherichia coli* (with 1 occurrence) (Figure 7) remained detectable for 7 days in stored irrigation water at 3 °C–11 °C and pH 7.5 (Machado-Moreira et al., 2021), and the enterohemorrhagic strain *E. coli O157:H7* (with 3 occurrences) (Figure 7) survived for 6 days at 20 °C in untreated irrigation water and extended its viability to 14 days at 4 °C in both untreated and sterilized irrigation systems (Van Der Linden et al., 2014), making it the second most surviving pathogen evaluated in irrigation water systems. *Listeria innocua* survived for 28 days under the same cool storage conditions (3 °C–11 °C, pH 7.5). Salmonella spp. occurred twice (Figure 6) in untreated irrigation water: surviving 6 days at 20 °C and 14 days at 4 °C, as reported by Van Der Linden et al. (2014). These two instances reflect moderate survival of *Salmonella* spp. across various environmental temperatures. *Salmonella typhimurium* (1 occurrence) also survives for 14 days in untreated irrigation water at both 4 °C and 20 °C (Van Der Linden et al., 2014). Collectively, these findings illustrate that, while all five pathogens can endure cold temperatures for at least 1 week, *E. coli O157:H7* and *Listeria innocua* show the longest survival times, reaching up to 14 and 28 days, respectively. The frequency of occurrences, ranging from one to three studies per pathogen, provides an overview of the relative research attention and documented resilience in irrigation water systems.

**TABLE 4:** Survival and persistence of pathogens in irrigation water systems.

Pathogen	Type	Water source	Duration	Condition	Reference
*E.coli*	Bacteria	Stored irrigation water	7 days	3 °C–11 °C pH 7.5	Machado-Moreira et al., 2021
*E. coli O157:H7*	Bacteria	Untreated irrigation water	6 days	20 °C	Van Der Linden et al., 2014
*E. coli O157:H7*	Bacteria	Untreated irrigation water	14 days	4 °C	Van Der Linden et al., 2014
*E. coli O157:H7*	Bacteria	Sterilized irrigation water	14 days	4 °C and 20 °C	Van Der Linden et al., 2014
*Listeria innocua*	Bacteria	Stored irrigation water	28 days	3 °C–11 °C pH 7.5	Machado-Moreira et al., 2021
*Salmonella* spp.	Bacteria	Untreated irrigation water	6 days	20 °C	Van Der Linden et al., 2014
*Salmonella* spp.	Bacteria	Untreated irrigation water	14 days	4 °C	Van Der Linden et al., 2014
*Salmonella typhimurium*	Bacteria	Untreated irrigation water	14 days	4 °C and 20 °C	Van Der Linden et al., 2014

### 3.8 Duration and frequency of pathogens in specialized and purified water systems

**FIGURE 8:**
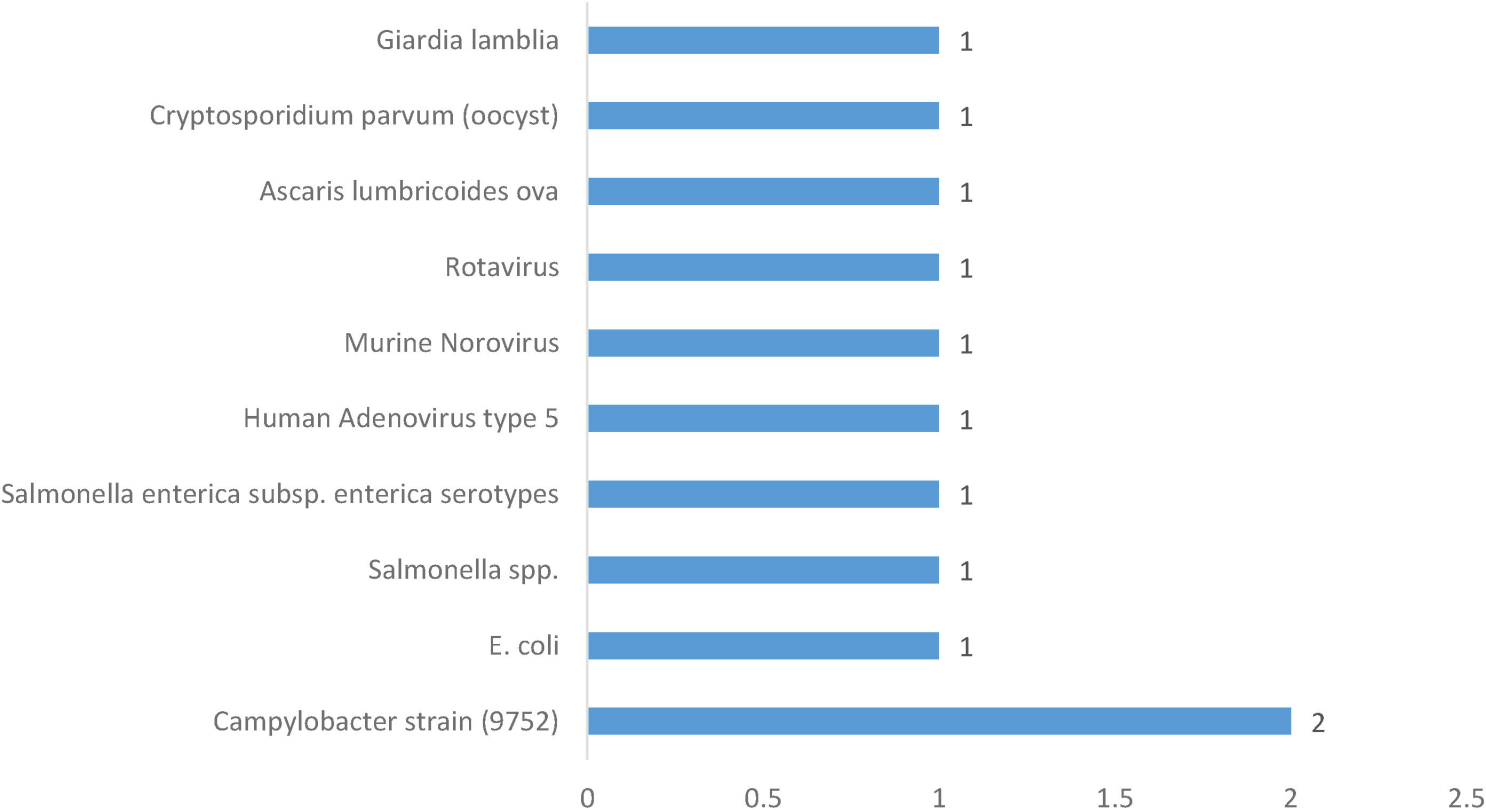
Frequency of pathogens in specialized/purified water systems.

*Campylobacter* strain 9752 is the most studied pathogen in a specialized water system with 2 occurrences (Figure 8). It has shown adaptability across temperature regimes, surviving for 360 h at 4 °C and for 72 h at 30 °C in two-stage sterilized water systems (Buswell et al., 1998; Table 5). In contrast, each of the other nine pathogens occurred only once in a specialized or purified water context (Figure 8). Among bacterial contaminants, *Escherichia coli* survived for 6 min in a reverse-osmosis water matrix undergoing lime stabilization at 28 °C and pH 11.5–12.0, demonstrating that even brief exposures to highly alkaline, purified water environments can sustain coliform viability under extreme chemical conditions (Bean et al., 2007). *Salmonella* spp. similarly, survived a 6-min exposure in the same reverse-osmosis, lime-stabilized matrix at 28 °C and pH 11.5–12.0, a comparable resistance among enteric bacteria to rapid water-treatment processes (Bean et al., 2007). Notably, *Salmonella enterica* subsp. *enterica* serotypes survived for an extended duration of 160 days in nuclease-free water at both 4 °C and 25 °C, revealing the long-term survival potential of this subspecies in purified water systems devoid of microbial competition or enzymatic degradation (Williams et al., 2024).

**TABLE 5:** Survival and persistence of pathogens in specialized or purified water systems.

Pathogen	Type	Water source	Duration	Condition	Reference
*Campylobacter* strain (9752)	Bacteria	Two-stage sterilized water biofilm	360 h	4 °C	Buswell et al., 1998
*Campylobacter* strain (9752)	Bacteria	Two-stage sterilized water biofilm	72 h	30 °C	Buswell et al., 1998
*E. coli*	Bacteria	Reverse osmosis water matrix (lime stabilization)	6 min	28 °C pH 11.5–12	Bean et al., 2007
*Salmonella* spp.	Bacteria	Reverse osmosis water matrix (lime stabilization)	6 min	28 °C pH 11.5–12.0	Bean et al., 2007
*Salmonella enterica* subsp. *enterica* serotypes	Bacteria	Nuclease-free water	160 days	4 °C, 25°C	Williams et al., 2024
Human adenovirus type 5	Virus	Reverse osmosis water matrix (lime stabilization)	6 min	28 °C, pH 11.5–12.0	Bean et al., 2007
Murine norovirus	Virus	Sterile demineralized water	>42 days	4 °C	Zhu et al., 2020
Rotavirus	Virus	Reverse osmosis water matrix (lime stabilization)	6 min	28 °C pH 11.5–12.0	Bean et al., 2007
*Ascaris lumbricoides ova*	Parasite	Reverse osmosis water matrix (lime stabilization)	72 h	28 °C pH 11.5–12.0	Bean et al., 2007
*Cryptosporidium parvum* (oocyst)	Parasite	Reverse osmosis water matrix (lime stabilization)	72 h	28 °C pH 11.5–12.0	Bean et al., 2007
*Giardia lamblia*	Parasite	Reverse osmosis water matrix (lime stabilization)	48 h	28 °C pH 11.5–12.0	Bean et al., 2007

Viral pathogens exhibited varied persistence profiles. Human Adenovirus type 5 maintained infectivity for 6 min in reverse-osmosis water with lime stabilization at 28 °C and alkaline pH, paralleling bacterial survival in the same treatment matrix (Bean et al., 2007). Murine norovirus demonstrated durability, surviving for over 42 days in sterile demineralized water at 4 °C, which may suggest that non-enveloped RNA viruses do survive in mineral-free, cold storage conditions (Zhu et al., 2020). Rotavirus also survived a 6-min exposure in the reverse-osmosis, lime-stabilized system at 28 °C and pH 11.5–12.0, and this further confirmed the capacity of diverse enteric viruses to withstand rapid treatment interventions (Bean et al., 2007).

Parasitic stages showed substantial resistance to specialized water systems as *Ascaris lumbricoides* ova and *Cryptosporidium parvum* oocysts each survived for 72 h in reverse-osmosis water with lime stabilization at 28 °C and pH 11.5–12.0 (Table 3). These could suggest the resilience of helminth eggs and protozoan cysts to remain viable under highly alkaline purification protocols (Bean et al., 2007). *Giardia lamblia* cysts persisted for 48 h under the same conditions, but slightly shorter, and still showed significant endurance of protozoan contaminants in treated water matrices (Bean et al., 2007). While rapid, chemically intensive purification processes can inactivate many pathogens within minutes, certain bacteria, viruses and parasites have shown they can endure brief exposures, and persist for months or weeks in specialized water systems (Table 5).

### 3.9 Mean survival and persistence of bacteria in water

Bacterial pathogens can survive and persist in different water bodies spanning for days, weeks, months and even years. Bacterial pathogens in water exhibited a mean survival duration of 28 days. Their persistence was significantly longer, with a mean duration of 621 days (Table 6).

**TABLE 6:** Average survival/persistence duration of bacteria.

Survival/persistence	Mean duration (days) ± SD	Range
Survival	28.29 ± 72.14	360–850
Persistence	621.00 ± 223.16	0.0042–750

#### 3.9.1 Survival and persistence durations of viruses

Viruses demonstrated an average survival duration of approximately 22 days, with a broad range from mere fractions of a day to as long as 70 days. In contrast, the persistence of certain viruses extended significantly, with a mean duration of 1095 days, suggesting resilience in aquatic environments

#### 3.9.2 Survival durations of parasites/protozoans

The study found that parasites/protozoans only showed survival, with an average duration of 30 days across different water sources, with a standard deviation of 57.31 days, indicating significant variability among different types of water sources, unlike bacteria and viruses, which showed both survival and persistence.

#### 3.9.3 Conditions influencing bacterial survival and persistence across different water system

##### 3.9.3.1 Influence of temperature on bacterial survival and persistence

Temperature is crucial in determining bacterial survival and persistence in various aquatic environments. Psychrophilic bacteria, which thrive in temperatures between 0 °C and 20 °C, exhibit the longest average survival duration of 57 days (Table 7). Several pathogens, including *Salmonella spp.*, *E. coli*, *Listeria monocytogenes*, and *Vibrio* species, persist for extended periods in cold water conditions. For instance, *Salmonella spp.* survives in unfiltered seawater for up to 32 weeks at 12 °C (Davidson et al., 2015), while *Campylobacter spp.* remain viable in water microcosms for 22–230 h at temperatures ranging from 4 °C to 37 °C (Buswell et al., 1998). Similarly, at 10 °C, *Listeria monocytogenes* survived for at least 47 and 28 days in drinking water with biofilm and without biofilm, respectively (Bjergbæk et al., 2021), while in seaweed-associated water, it survived for 7 days at both 4 °C and 10 °C (Akomea-Frempong et al., 2023). Psychrotrophic bacteria, which can grow at temperatures exceeding 20 °C but below 30 °C, demonstrate an average survival duration of 39 days (Table 8). *E. coli* strains, including *O157:H7*, remain viable in freshwater environments at 18 °C or 20 ± 2 °C for more than 42 days (Martín-Díaz et al., 2017), whereas *Salmonella* spp survived for 8 h at 22 °C in seaweed-associated water (Akomea-Frempong et al., 2023) and for 5 and 14 days at 8 °C and 21 °C, respectively in bovine faeces water (Amalaradjou et al., 2006). Mesophilic bacteria, which thrive at temperatures between 30 °C and 45 °C, exhibit a slightly lower average survival duration of 36 days (Table 8). Some bacteria, such as *Salmonella typhimurium* and *Campylobacter* spp, persist in marine and freshwater environments within this temperature range Wang et al., 2018). Thermophilic bacteria, which thrive at temperatures between 45 °C and 80 °C, show the shortest survival duration, averaging less than a day. This could suggest that high temperatures significantly reduce bacterial viability, limiting the persistence of most pathogens in warmer conditions, but they persist longer in colder environments, particularly within the psychrophilic and psychrotrophic temperature ranges. This highlights the potential for extended bacterial contamination in water systems exposed to lower temperatures, posing risks for waterborne disease transmission. Conversely, higher temperatures generally reduce bacterial survival, suggesting that thermal conditions may be a limiting factor for bacterial survival and persistence in warmer aquatic environments.

**TABLE 7:** Bacteria survival and persistence across temperature categories.

Temperature category	Average duration (days)
Psychrophiles (0 °C–20 °C)	56.87
Psychrotrophs (>20 °C–30 °C)	39.10
Mesophiles (>30–45 °C)	36.00
Thermophiles (>45 °C–80 °C)	0.33
Unknown	32.27

**TABLE 8:** Average survival/persistence duration of viruses.

Survival/persistence	Mean duration (days) ± SD	Range
Survival	22.48 ± 19.44	0.004–70
Persistence	1095 ± 0.00	1095

##### 3.9.3.2 Influence of pH on bacterial survival and persistence

Bacteria tend to survive much longer in water environments with near-neutral or slightly alkaline pH, whereas extreme pH conditions can curtail their survival and persistence dramatically. *Escherichia coli* shows that when present in river water, with pH consistently reported in the range of 7.4–8.0, the bacteria can survive for 11 days (Martín-Díaz et al., 2017). In one study, *E. coli* remained viable for as long as 26 days (Ballesté et al., 2024) under controlled laboratory culture conditions, and in another instance, using qPCR methods, survival extended to over 42 days (Martín-Díaz et al., 2017). This prolonged survival may suggest that the near-neutral pH typical of many natural water bodies provides a stable environment that supports bacterial cell integrity and metabolic function. In contrast, extreme alkaline conditions show a starkly different picture. *E. coli* (*O157:H7*) in reverse osmosis water, in which the water matrix reportedly had a pH near 12, demonstrated that the pathogen survived for only 6 min (Bean et al., 2007). Such a rapid decline in viability under highly alkaline conditions may explain the sensitivity of bacterial cells to growth environments that are far removed from their optimum pH ranges. A similar pattern is observed with stored irrigation water at a controlled pH of 7.5. *E. coli* survived for 7 days (Machado-Moreira et al., 2021), and this further reinforces how neutral pH levels favor bacterial endurance. The acidic environment tends to favor *E. coli* survival. In untreated river water at a pH of 2.5–3.0, *E. coli* survived for 109 days (Scott et al., 2006).

#### 3.9.4 Conditions influencing viral survival and persistence in water

##### 3.9.4.1 Influence of temperature on viral survival and persistence

Viral pathogens at low and refrigerated temperatures, which thrive at temperatures between 0 °C and 20 °C, exhibited an average survival duration of 21 days (Table 9), with notable resilience seen in viruses such as Avian influenza and several types of polioviruses. In contrast, room temperature viral pathogens (20 °C–30 °C) demonstrated a reduced survival duration average of 13 days. This may explain the impact of slightly elevated temperatures on viral viability. Viral pathogens found at temperatures above 30 °C showed a marked increase in survival, with an average of 42 days (Table 2).

**TABLE 9:** Viral survival and persistence across temperature categories.

Temperature category	Average duration (days)	Virus	References
Low and refrigerated temperature (0 °C–20 °C)	21.45	Avian influenza virus (LPAIV) HINI Human adenovirus (4041) Infectious poliovirus type 1 (genome) Murine norovirus Poliovirus (MS2) Poliovirus (x174), Poliovirus genome SARS-CoV	McQuaig et al., 2009; Perlas et al., 2023; Helmi et al., 2008; Zhu et al., 2020; Contrant et al., 2023; Basavaraju et al., 2021
Roomtemperature (>20 °C–30 °C)	13.14	Human adenovirus Human adenovirus (4041) Human adenovirus type 5 Human polyomaviruses Murine norovirus Rotavirus Virulent *Aeromonas hydrophila* (vAh)	McQuaig et al., 2009; Tuttle et al., 2023; Hsueh and Gibson, 2015; Contrant et al., 2023
Warm (>30)	42	Human adenovirus Human Norovirus Human polyomaviruses	McQuaig et al., 2009; Anderson et al., 2011

##### 3.9.4.2 Influence of pH on viral survival and persistence

Rotavirus persisted in a reverse osmosis water matrix for only 6 min at a high pH range of 11.5–12.0 and a temperature of 28 °C (Bean et al., 2007). Similarly, Adenovirus was tested under the same pH conditions (11.5–12.0) in a reverse osmosis water matrix, where it survived for only 6 min (Bean et al., 2007). However, in filtered and unfiltered water at neutral pH and lower temperatures (15 °C and 25 °C), adenovirus persisted for up to 28 days (Ahmed et al., 2021). These survival times show that extreme pH values may accelerate viral degradation, while a more neutral pH environment allows for prolonged persistence. Poliovirus also exhibited pH sensitivity, particularly in drinking water biofilms. At a slightly alkaline pH of 9.5, poliovirus persisted for up to 6 days at 10 °C, but when monitored under the same pH conditions, it remained viable for up to 34 days using qPCR detection (Helmi et al., 2008). This extended survival suggests that poliovirus is more resistant to moderate alkaline conditions than rotavirus and adenovirus, reinforcing the idea that different viruses have unique pH tolerances.

#### 3.9.5 Conditions influencing parasites/protozoans’ survival and persistence in water

##### 3.9.5.1 Influence of temperature on parasites/protozoans’ survival and persistence

*Trichobilharzia szidati cercariae* showed a progressive decrease in survival duration as temperature increased, surviving for 204 h at 5 °C but only 36 h at 30 °C (Al-Jubury et al., 2020). This suggests that colder environments may facilitate the prolonged infectivity of certain parasites, posing a greater risk in cooler water bodies. Similarly, *Colpoda spp.* and *Naegleria spp.* exhibited extended survival in colder conditions, with survival reaching 6 months in temperatures ranging from 3 °C to 21 °C (Baré et al., 2011). The ability of these protozoans to endure for months in underground or chlorinated water further exacerbates the potential for long-term contamination in such environments.

##### 3.9.5.2 Influence of pH on parasites/protozoans survival and persistence

pH variations also influence parasite survival, with alkaline conditions sometimes extending persistence. *Cryptosporidium parvum* and *Giardia lamblia* survived in reverse osmosis water matrices at pH 11.5–12.0 for 72 and 48 h (Bean et al., 2007), respectively. While extreme pH levels can limit some bacterial and viral pathogens, certain protozoans can tolerate and survive in highly alkaline environments.

### 3.10 Risk of bias

**FIGURE 9:**
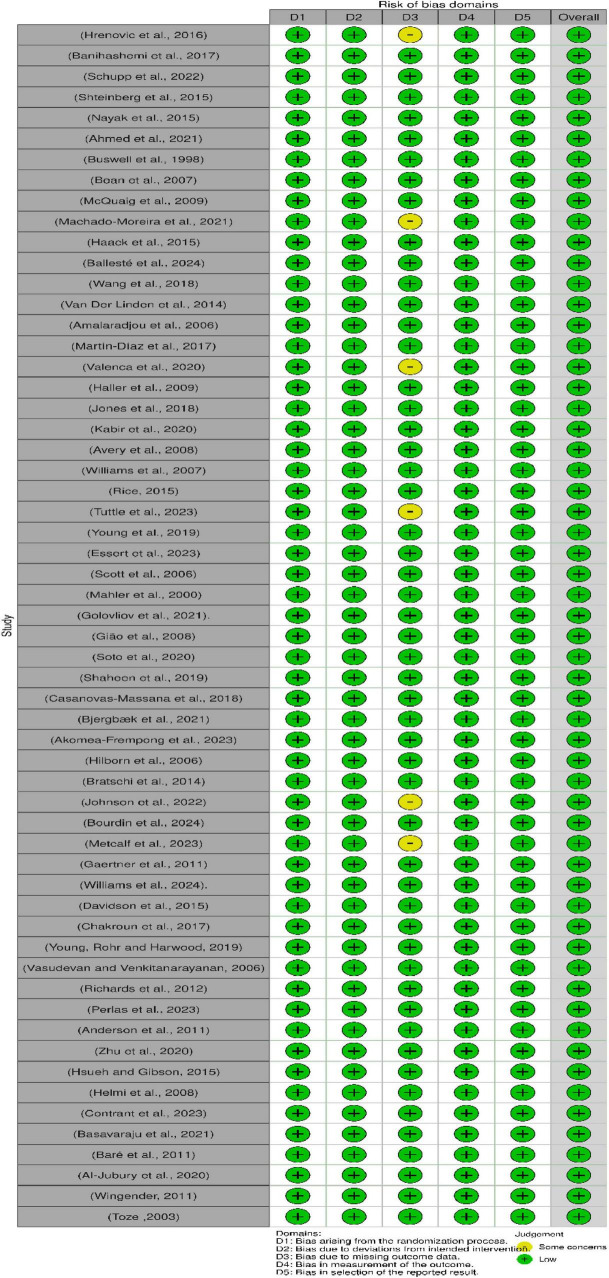
Assessment of bias in the included studies via the Cochrane risk of bias tool. D1: Bias arising from the randomization process, D2: Bias due to deviations from intended intervention, D3: Bias due to missing outcome data, D4: Bias in measurement of the outcome, D5: Bias in selection of the reported result.

The risk of bias for the 58 studies included in this review was assessed via the Robvis tool, as shown in Figure 9. The tool categorizes bias into three levels: low risk (green), some concern (yellow), and high risk (red). The overall low risk of bias across all the domains suggested that the studies were methodologically sound and reliable.

## 4 Discussion

The persistence of pathogenic microorganisms in various water types and sources is a critical concern for public health, environmental monitoring, and water management. This review synthesizes data from a range of studies on the survival and persistence of different groups of pathogens, including bacteria, viruses, parasites, and fungi, in diverse water types, such as freshwater, seawater, sewage, irrigation water and purified water, under various external conditions like temperature, pH, and the presence of organic matter. This review also includes the various detection methods used to identify the pathogens in the various water sources. The findings in this review have broad implications in the context of waterborne disease transmission, antimicrobial resistance, and environmental health.

### 4.1 Pathogens and freshwater system

Pathogen survival in freshwater spans from days to years, driven by temperature, pH, nutrient availability, and interaction with particulates. *Escherichia coli* and *Salmonella* survive up to 90 days when associated with sediment microcosms, compared to markedly shorter planktonic lifespans. Freshwater sediments provide multifaceted protection for pathogenic microorganisms, significantly enhancing their survival and persistence in aquatic environments. Association with sediment particles offers pathogens critical protection from UV radiation, which is a primary inactivating factor in the water column. The particulate material acts as a physical shield, preventing harmful solar radiation that would otherwise cause DNA damage and inactivation of bacterial and viral pathogens. Additionally, sediment-associated pathogens experience enhanced protection from predation by protozoa and other microorganisms (Wu et al., 2005; Malham et al., 2014). Norovirus and human adenovirus maintain infectivity for over 70 days in surface water and for years in groundwater, challenging the assumptions that open-system waters rapidly inactivate enteric viruses (Kennedy et al., 2023).

### 4.2 Marine water systems

High salinity exerts strong osmotic pressure that accelerates the die-off of classic enteric bacteria such as Escherichia coli, yet halotolerant species like Salmonella spp. and *Pseudomonas aeruginosa* survive for months by accumulating compatible solutes (e.g., trehalose, glycine betaine) and remodeling membrane lipids to maintain cell turgor. Under saline stress combined with antagonistic indigenous microbiota, fecal indicator bacteria decline rapidly, often falling below detectable levels within days. In contrast, enteric viruses (norovirus, poliovirus) and protozoan cysts endure for extended periods by adsorbing to marine sediments or embedding within biofilms, where organic matter and extracellular polymeric substances protect viral capsids and oocyst walls from inactivation. While lower temperatures slow inactivation kinetics across all pathogens, high salinity accelerates bacterial decay, effectively selecting pathogens with adaptive persistence mechanisms. These calls for marine-specific risk assessments must integrate salinity, temperature, sediment associations, and biotic interactions to accurately predict pathogen fate in coastal waters (Wingender, 2011; Schupp et al., 2022).

### 4.3 Sewage water systems

Enteric bacteria and viruses demonstrate endurance in sewage matrices, outliving their persistence in freshwater and marine environments. Uropathogenic and intestinal *Escherichia coli* strains have been shown to survive through every stage of sewage treatment, including disinfection, and identical clones have been detected in downstream receiving waters up to 20 km from wastewater treatment plants, which shows their release and environmental persistence beyond treated effluent discharge points (Anastasi et al., 2012). *Salmonella enterica* exhibits even greater longevity in controlled laboratory settings, where it remained viable for up to 5 years in sterile water at 25 °C, and field studies have documented survival periods extending beyond 231 days in manure-amended waters (Liu et al., 2018), emphasizing the role of organic-rich sewage in sustaining this pathogen far longer than the typical less than 30 days observed in fresh surface waters or the less than 10 days reported in saline marine systems. Human adenoviruses, frequently detected at concentrations exceeding 10^6^ viral genomes per liter in raw sewage, retain infectivity for months, with T-90 values for 90% reduction of approximately 153 days at 4 °C and 38 days at 23 °C (Fong et al., 2010), surpassing their decay rates in groundwater and surface waters and this could explain the role of sewage as a reservoir for prolonged viral shedding into the environment.

### 4.4 Irrigation water systems

In irrigation water systems, pathogens such as *E. coli* O157:H7, *Salmonella* spp., and *Listeria innocua* demonstrate a capacity for prolonged survival that, under certain conditions, matches or even exceeds their persistence in freshwater, marine, and sewage environments. Peaked at low temperatures (4 °C), *E. coli* O157:H7 has been shown to survive for up to 14 days in both untreated and sterilized irrigation microcosms, nearly doubling its viability compared to warmer conditions, a trend consistent with findings that cold storage slows metabolic inactivation and nutrient depletion, thereby lengthening bacterial persistence (Pierre and Christner, 2009). This cryoprotection is further accentuated by the ability of *E. coli* O157:H7 to enter into protective associations with free-living protozoa such as *Acanthamoeba polyphaga*, effectively using these hosts as reservoirs for extended survival in irrigation environments. *Salmonella* spp., meanwhile, can endure a broad pH range (4.0–9.5) and survive for up to 30 days in surface waters used for irrigation, often persisting in biofilms or within sediments where they are shielded from UV radiation and predation by indigenous microbiota (Liu et al., 2018). The resilience of *Listeria* spp. parallels that of enteric bacteria; in irrigation water spiked with 10^7^ CFU/mL, *L. innocua* remained detectable by culture and qPCR for up to 28 days, which suggest its potential to contaminate produce through stored irrigation water systems (Machado-Moreira et al., 2021). Notably, the survival durations in irrigation water are 7 days for generic *E. coli* at 20 °C and 3 °C–11 °C, 14 days for *Salmonella* spp. at 4 °C, and 28 days for *L. innocua* surpass those reported for marine systems (maximum 7 days for *E. coli* in tidal creeks) and are comparable to, or exceed, survival in raw sewage at ambient temperatures (over 28 days at 25 °C).

### 4.5 Purified water

The extended persistence of pathogens in highly purified or specialized water systems, despite conditions engineered to inactivate microorganisms. Purified water matrices (e.g., demineralized, nuclease-free, or reverse-osmosis water) cannot guarantee pathogen elimination through chemical treatment alone. Non-enveloped viruses (e.g., norovirus) and protozoan cysts maintain infectivity for weeks to months under sterile, nutrient-free conditions at low temperatures. Surface interactions within piping networks further shelter pathogens like *Acinetobacter baumannii* in biofilms, indicating that system design must integrate physical cleaning, chemical disinfectants, and temperature control to prevent recolonization and downstream contamination.

### 4.6 Environmental drivers of pathogen survival and persistence

Temperature is one of the most influential environmental factors affecting pathogen survival and persistence. Generally, lower temperatures enhance the survival of pathogens by slowing down metabolic activity and degradation processes. Psychrophilic bacteria like *Salmonella spp.* and *Listeria monocytogenes* exhibit extended survival in cold conditions, persisting for weeks to months in water at temperatures below 20 °C (Liu et al., 2018). Similarly, viruses such as noroviruses and adenoviruses have been reported to survive for years in groundwater under low-temperature conditions (Toze, 2003). In contrast, higher temperatures accelerate pathogen die-off by increasing enzymatic activity and microbial competition. For example, thermophilic bacteria like *Vibrio parahaemolyticus* survive for less than a day in water exceeding 45 °C (Dekic et al., 2018). Mesophilic Mycoplasma bovis survived for 6 hours in shaded water at about 15 °C and but declined to 2 hours at 19 °C when the water was exposed to sunlight (Johnson et al., 2022). However, other mesophilic pathogens like *E. coli O157:H7* can adapt to a broader temperature range, surviving for over 42 days in freshwater at moderate temperatures (18 °C–20 °C) (El-Sharkawi et al., 1989).

In addition to the impact of temperature, pH also played a role in shaping the persistence of various pathogens. Neutral to slightly alkaline conditions (pH 7–8) are generally favorable for bacterial survival. Extreme pH levels, however, are detrimental to most pathogens. Highly alkaline conditions (pH > 11) rapidly inactivate bacteria like *E. coli*, which survive for only 6 min at pH 12 (Dekic et al., 2018). Even though some viruses can persist in harsh environments, their behavior can be altered by pH levels. Subjecting viruses to extremely alkaline conditions (pH above 11) can interfere with their capsid and envelope structures, resulting in the denaturation and disintegration of the viral particles (Seguchi et al., 2024; Gerba et al., 2024). The rapid inactivation of viruses at these pH levels can be an important pre-treatment or supplementary step to complement other disinfection methods. However, the protozoan parasites *Ascaris lumbricoides, Giardia lamblia* and *Cryptosporidium parvum* can survive a high alkaline water environment. *Ascaris lumbricoides* and *Cryptosporidium parvum* persisted for 72 h and *Giardia lamblia* for 48 h, respectively, in an alkaline water environment at a pH of 11.5–12.0 (Bean et al., 2007). *Cryptosporidium parvum* is a highly resistant pathogen, capable of forming protective oocysts that can withstand various environmental stresses. The ability of these protozoans to persist in a highly alkaline water environment is concerning, as it suggests that high pH alone may not be sufficient to effectively inactivate this pathogen, emphasizing the need for complementary disinfection strategies alongside pH adjustments.

The review also revealed that various water systems can still harbor pathogens for extended periods, particularly in protective biofilms, even after treatment processes, such as chlorination. *Legionella pneumophila* have been found to persist in drinking water for up to 850 days (Shaheen et al., 2019), while *Listeria monocytogenes* and *Helicobacter pylori* survived for up to 47 days and 31 days in drinking water biofilm, respectively (Hrenovic et al., 2016; Gião et al., 2008). These biofilm-associated pathogens are particularly concerning because they can be released into the water supply system, potentially leading to outbreaks of waterborne disease. The persistence of these pathogens in treated water is concerning, as chlorinated water is often assumed to be free of pathogens and may be used for drinking, bathing, and recreational activities. This calls for the need for complementary disinfection strategies. Water management authorities must adopt a proactive, multi-barrier approach that combines effective treatment technologies, continuous monitoring, and swift response mechanisms to address the persistent nature of pathogens in water systems. UV irradiation and ozonation, for example, can enhance the effectiveness of water treatment and reduce the risk of pathogen survival or biofilm formation (Tsydenova et al., 2015; Kalisvaart, 2004). Furthermore, exploring innovative approaches, such as combined disinfectants, advanced oxidation processes, or integrating physical and biological treatment barriers, may improve the elimination of persistent pathogens in water.

### 4.7 Survival and persistence duration of bacteria, viral, parasites and fungi

The ability of some bacterial pathogens to form and survive in biofilms, in nutrient-rich and nutrient-poor environments, likely contributes to their persistence across the various water sources in the study (Haack et al., 2015; Van Der Linden et al., 2014). In contrast, viruses are more susceptible to environmental changes, such as pH and temperature fluctuations (Labadie et al., 2018), and their detection and quantification in environmental samples are more complex and resource-intensive, leading to a relatively smaller body of research compared to bacteria. Furthermore, there are methodological challenges in quantifying viruses in water sediments, including poor recovery rates and the inability to distinguish between infective and non-infective particles, which contribute to underestimating viral presence (Sooryanarain and Elankumaran, 2015; Zhang et al., 2024).

Parasites, despite demonstrating survival, especially in dechlorinated tap water (Al-Jubury et al., 2020), chlorinated water (Baré et al., 2011), and biofilms (Helmi et al., 2008), are less frequently studied, potentially due to the complexity of their life cycles and sporadic occurrence in water sources (Mthethwa et al., 2021). Fungi, the least represented in the literature, have only recently gained recognition as potential waterborne pathogens, particularly in immunocompromised individuals (Genet and Girma, 2024), which may explain the limited research in this area. However, studies have investigated the survival of human pathogenic fungi in water sources. For example, a study demonstrated that dermatophyte fungi could survive in chlorinated swimming pool water for up to 123 days (Fischer, 1982). Similarly, a study found that propagules of four species of human pathogenic fungi (*Trichophyton mentagrophytes*, *Trichosporon cutaneum*, *Candida albicans*, and *Microsporum gypseum*) remained viable in seawater for up to 52 weeks, with persistence based on varying temperature (Anderson, 1979). In another study, several species of *Trichophyton*, *Microsporum*, and *Chrysosporium* fungi remained intact for 7–10 years in sterile water without any noticeable degradation (Deshmukh, 2003). The survival of fungi responsible for agricultural diseases (Johnson and Speare, 2003) has also been well-documented. A recent study on the Fusarium wilt pathogen, *Fusarium oxysporum* f. sp. cubense, found that its spores could remain viable in water for over 120 days (Ullah et al., 2021). Similarly, the fungus responsible for amphibian chytridiomycosis, Batrachochytrium dendrobatidis, has been shown to retain infectivity in lake water for up to 7 weeks, underscoring the risk it poses to aquatic ecosystems. Therefore, expanding research on fungal pathogen survival in diverse water sources and evaluating integrated disease management strategies are crucial to mitigate the risks of waterborne disease transmission and spread.

Moving forward, a concerted effort is needed to expand the research on pathogen survival in the environment. This should involve the adoption of more holistic, multi-disciplinary approaches that integrate laboratory-based studies with field-based investigations. One potential avenue for future research is using advanced analytical techniques to better understand the mechanisms and factors influencing pathogen persistence in different environmental matrices. The application of molecular methods, such as quantitative PCR or next-generation sequencing, could provide insights into the population dynamics and gene expression profiles of pathogens under various environmental conditions (Sajid et al., 2023; Charron et al., 2025). This could help elucidate these microorganisms’ physiological adaptations and survival strategies. Additionally, the incorporation of environmental modeling and simulation approaches may be valuable in predicting the dispersal, transport, and fate of pathogens in complex, real-world systems (Bradford et al., 2014). By integrating data on abiotic factors (e.g., temperature, humidity, water chemistry), biotic interactions (e.g., microbial communities, predation), and anthropogenic influences (e.g., water management, land use), researchers could develop more robust and realistic models to inform disease control efforts.

While this review followed the PRISMA guidelines and consolidated extensive research on pathogen persistence in water, it has several limitations. The apparent disparity in the research volume on different pathogen types, with bacteria being the most extensively studied, results in less comprehensive data on viruses, parasites, and fungi. The choice of detection method also significantly impacts the reported persistence of pathogens. While widely used, culture-based methods often underestimate pathogen viability, particularly for organisms in a viable but non-culturable (VBNC) state. However, reliance on molecular methods raises questions about the practical implications of detecting non-viable pathogens, emphasizing the need for complementary approaches that assess infectivity. This skews the broader impact of the review’s conclusions, particularly regarding the persistence of non-viable pathogens. Additionally, the influence of various environmental factors, such as temperature and water type, introduces variability in the results, making it challenging to generalize the findings across different settings. The review also highlights the challenges in detecting and quantifying specific pathogens, particularly viruses, which are resource-intensive to study and thus less frequently researched. Finally, the reliance on controlled laboratory settings in many studies may not fully capture the complexity and diversity of real-world water systems, limiting the review’s applicability to natural water environments.

## 5 Conclusion

This systematic review highlights the importance of understanding pathogen survival and persistence in water for public health and advancing water industry practices. The survival and persistence of bacteria, viruses, parasites, and fungi in diverse aquatic environments highlight the potential for waterborne disease transmission and the limitations of current water treatment strategies. Robust surveillance systems are needed to monitor pathogen presence in water sources, especially in regions with inadequate sanitation or high-risk environmental conditions. This knowledge is crucial for developing proactive prevention strategies, such as optimizing disinfection protocols and implementing multi-barrier approaches to ensure water safety.

Research gaps, particularly regarding the survival mechanisms of parasites and fungi, are also highlighted. Addressing these gaps is crucial for enhancing our understanding of pathogen persistence and improving water safety research. Future studies should prioritize investigating lesser-studied pathogens to inform more comprehensive risk assessments and mitigation strategies. Integrating multiple scientific disciplines, such as genomics, environmental science, and microbiology, offers a promising avenue for advancing research on pathogen survival.

The research community must adopt a more coordinated approach to address persistent pathogens in water systems, exploring interactions between pathogens and biofilms, evaluating the efficacy of advanced disinfection technologies, and developing predictive models to assess pathogen risks under changing environmental conditions.

## Data Availability

The original contributions presented in this study are included in this article/Supplementary material, further inquiries can be directed to the corresponding authors.
