# Supplementary material for: How long do pathogens persist and survive in water? A systematic review
Source: Front Microbiol. 2025 Oct 28;16:1654785. doi: 10.3389/fmicb.2025.1654785 (PMC12602397; doi:10.3389/fmicb.2025.1654785)
Supplement: Supplementary file 1 [file Table_1.docx]

**Table S1.** Survival and persistence of bacteria in various water types/sources

| **Bacteria** | **Water Source** | **Duration** | **Condition** | **Method of Detection** | **Persistence/Survival** | **Reference** |
| --- | --- | --- | --- | --- | --- | --- |
| *Acinetobacter baumannii* | Sterilized effluent wastewater | 50 days | _ | Culture | Survival | (Hrenovic et al., 2016) |
| *Arcobacter butzleri* | Sterile and non-sterile river water | 28 days | (15 & 25) °C | qPCR | Survival | (Banihashemi et al., 2017) |
| *Bacillus globigii* (surrogate for *Bacillus anthracis*) | Waste water | At least 50 days | 20-27°C | Culture | Survival | (Schupp et al., 2022) |
| *Burkholderia cenocepacia* | Atlantic Ocean | 1 month | 20°C | Culture | Survival | (Shteinberg et al., 2015) |
| *Burkholderia cenocepacia* | Dead sea | 24 hours | 20°C | Culture | Survival | (Shteinberg et al., 2015) |
| *Campylobacter jejuni* | Sterile and non-sterile river water | 28 days | 15°C,25°C | qPCR | Survival | (Banihashemi et al., 2017) |
| *Campylobacter jejuni* | Fresh water and Marine surface water | 2 days | 22°C and 27°C | qPCR | Survival | (Nayak et al., 2015) |
| *Campylobacter* spp. | Filtered & unfiltered water | 28 days | 15°C,25°C | qPCR | Survival | (Ahmed et al., 2021) |
| *Campylobacter strain(9752)* | Water microcosm | 157 hours | 4°C | Immunofluorescent- antibody staining | Survival | (Buswell et al., 1998) |
| *Campylobacter strain(9752)* | Water microcosm | 108 hours | 10°C | Immunofluorescent- antibody staining | Survival | (Buswell et al., 1998) |
| *Campylobacter strain(9752)* | Two-stage sterilized water biofilm | 360 hours | 4°C | Immunofluorescent- antibody staining | Survival | (Buswell et al., 1998) |
| *Campylobacter strain(9752)* | Two-stage sterilized water biofilm | 72 hours | 30°C(anaerobic) | Immunofluorescent- antibody staining | Survival | (Buswell et al., 1998) |
| *Campylobacter strain(9752)* | Two-stage sterilized water biofilm | 264 hours | 30°C(microaerobic) | Immunofluorescent- antibody staining | Survival | (Buswell et al., 1998) |
| *Campylobacter strain(CH1&9752)* | Water microcosm | 202 hours(mean) | 4°C | Immunofluorescent- antibody staining | Survival | (Buswell et al., 1998) |
| *Campylobacter strain(CH1&9752)* | Water microcosm | 176 hours(mean) | 10°C | Immunofluorescent- antibody staining | Survival | (Buswell et al., 1998) |
| *Campylobacter strain(CH1&9752)* | Water microcosm | 43 hours | 22°C | Immunofluorescent antibody staining | Survival | (Buswell et al., 1998) |
| *Campylobacter strain(CH1&9752)* | Water microcosm | 22 hours | 37°C | Immunofluorescent- antibody staining | Survival | (Buswell et al., 1998) |
| *Campylobacter strain(CH1)* | Water microcosm | 230 hours | 4°C | Immunofluorescent antibody staining | Survival | (Buswell et al., 1998) |
| *Campylobacter strain(CH1)* | Two-stage sterilised water biofilm | 700 hours | 4°C | Immunofluorescent antibody staining | Survival | (Buswell et al., 1998) |
| *Campylobacter strain(CH1)* | Two-stage sterilised water biofilm | 144 hours | 30°C(anaerobic) | Immunofluorescent antibody staining | Survival | (Buswell et al., 1998) |
| *Campylobacter strain(CH1)* | Two-stage sterilised water biofilm | 96 hours | 30°C(microaerobic) | Immunofluorescent antibody staining | Survival | (Buswell et al., 1998) |
| *E. coli* | Seawater and freshwater are submerged with microplastic and glass particles | >27 days | 15°C | Culture | Survival | (Metcalf et al., 2023) |
| *E. coli* | Reverse osmosis water matrix (lime stabilisation) | 6 minutes | 28°C  pH 11.5-12 | Most probable number (Culture) | Survival | (Bean et al., 2007) |
| *E. coli* | Sewage water | 28 days | 25 and 35°C | qPCR | Survival | (McQuaig et al., 2009) |
| *E. coli* | Stored irrigation water | 7 days | 3-11°C  pH 7.5 | Culture and qPCR | Survival | (Machado-Moreira et al., 2021) |
| *E. coli* | Stream water | 25 days | 18°C | qPCR | Survival | (Haack et al., 2015) |
| *E. coli* | Sea water | 7 days | 20 ± 2°C  pH 8.0 ± 0.1. | Culture | Survival | (Ballesté et al., 2024) |
| *E. coli O157:H7* | Microcosms with stream water | >6 hours | 50°C (free-floating) | Culture | Survival | (Wang et al., 2018) |
| *E. coli O157:H7* | Microcosms with stream water | >24hours | 50°C (sediment particle attached) | Culture | Survival | (Wang et al., 2018) |
| *E. coli O157:H7* | Microcosms with stream water | >1.7 hours | 60°C (sediment particle attached) | Culture | Survival | (Wang et al., 2018) |
| *E. coli O157:H7* | Microcosms with stream water | <1.5 hours | 60°C (free-floating) | Culture | Survival | (Wang et al., 2018) |
| *E. coli O157:H7* | Untreated irrigation water | 6 days | 20°C | Culture | Survival | (Van Der Linden et al., 2014) |
| *E. coli O157:H7* | Water without faeces or feed | 14 days | 21°C (75 mM sodium capyrate) | Culture | Survival | (Amalaradjou et al., 2006) |
| *E. coli O157:H7* | Water without faeces or feed | 5 days | 21°C (100 mM sodium capyrate) | Culture | Survival | (Amalaradjou et al., 2006) |
| *E. coli O157:H7* | Water without faeces or feed | 3 days | 21°C, 8°C (120 mM sodium capyrate) | Culture | Survival | (Amalaradjou et al., 2006) |
| *E. coli O157:H7* | Water with 1% bovine faeces | 5 days | 21°C, (120 mM sodium capyrate) | Culture | Survival | (Amalaradjou et al., 2006) |
| *E. coli O157:H7* | Water without faeces or feed | 14 days | 8°C (100 mM sodium capyrate) | Culture | Survival | (Amalaradjou et al., 2006) |
| *E. coli O157:H7* | Water with 1% bovine faeces | 21 days | 8°C and 21°C (75, 100 and 120mM sodium capyrate) | Culture | Survival | (Amalaradjou et al., 2006) |
| *E. coli O157:H7* | Water with 1% cattle feed | 24 hours | 8°C (75,100 and 120-mM sodium capyrate) | Culture | Survival | (Amalaradjou et al., 2006) |
| *E. coli O157:H7* | Water with 1% cattle feed | 24 hours | 21°C (75, 100 and 120nM sodium capyrate) | Culture | Survival | (Amalaradjou et al., 2006) |
| *E.coli* | River water | 11 days | 20 ± 2°C  pH 7.4 - 8.0 | Culture | Survival | (Martín-Díaz et al., 2017) |
| *E.coli* | River water | >42 days | 20 ± 2°C  pH 7.4 - 8.0 | qPCR | Survival | (Martín-Díaz et al., 2017) |
| *E.coli* | Sea water and freshwater submerged with microplastic and glass particles | >14 days | 15°C | Culture | Survival | (Metcalf et al., 2023) |
| *E.coli* | Sea water | 26 days | 20 ± 2°C  pH 8.0 ± 0.1. | qPCR | Survival | (Ballesté et al., 2024) |
| *E.coli* | River water | > 42 days | 20 ± 2°C | qPCR | Survival | (Martín-Díaz et al., 2017) |
| *E.coli* | Stormwater water | 15 days | 37°C (wildfire residues) | Culture | Survival | (Valenca et al., 2020) |
| *E.coli* | Storm water | >15 days | 37°C (unburned soil particles | Culture | Survival | (Valenca et al., 2020) |
| *E.coli* | Fresh water sediment | 90 days | _ | Culture | Survival | (Haller et al., 2009) |
| *E.coli* | Lake water microcosm | 28 days | 15°C,25°C | Culture | Survival | (Ahmed et al., 2021) |
| *E.coli (eaeA gene)* | Tidal creek | 10-17 days | 4.46-38.56°C | qPCR | Survival | (Jones et al., 2018) |
| *E.coli (set1B gene)* | Tidal creek | 3 months | 4.46-38.56°C | qPCR | Survival | (Jones et al., 2018) |
| *E.coli DNA* | Sea water | 26 days | 20°C (plastic pellets in seawater) | Culture and qPCR | Survival | (Ballesté et al., 2024) |
| *E.coli O157:H7* | Untreated irrigation water | 14 days | 4°C | Culture | Survival | (Van Der Linden et al., 2014) |
| *E.coli O157:H7* | Sterilized irrigation water | 14 days | 4°C & 20°C | Culture | Survival | (Van Der Linden et al., 2014) |
| *E.coli O157:H7* | Sterilized River water | 28 days | 5, 25 & 37°C | Culture | Survival | (Kabir et al., 2020) |
| *E.coli O157:H7* | Non sterile lake & river microcosm | 64 days | 10°C | Culture | Survival | (Avery et al., 2008) |
| *E.coli O157:H7* | Sterile puddled water  microcosms | 64 days | 10°C | Culture | Survival | (Avery et al., 2008) |
| *E.coli O157:H7* | Non-sterile puddled water  microcosms | At least 18 days | 10°C | Culture | Survival | (Avery et al., 2008) |
| *E.coli O157:H7* | Seawater | 5 days | 10 ± 0.1 °C | Culture | Survival | (Williams et al., 2007) |
| *Francisella tularensis subsp. holarctica NY98* | Sterile tap water | 28 days | 8°C | Culture | Survival | (Rice, 2015) |
| *Francisella tularensis subsp. holarctica NY98* | Filter-sterilised brook water | At most 10 days | 4°C | Culture | Survival | (Rice, 2015) |
| *Francisella tularensis subsp. holarctica NY98* | Pond water | 31 days | - | Animal infectivity assays | Survival | (Rice, 2015) |
| *Francisella tularensis subsp. holarctica  LVS  strain* | Sterile tap water | 21 days | 8°C | Culture | Survival | (Rice, 2015) |
| *Edwardsiella ictaluri* | Pond sediments | 14 days | 27.5 ± 0.3 °C | Culture | Survival | (Tuttle et al., 2023) |
| *Enterococci* | Sewage water | 28 days | 25 and 35°C | qPCR | Survival | (McQuaig et al., 2009) |
| *Enterococcus faecalis* | Sea water and freshwater submerged with microplastic and glass particles | >27 days | 15°C | Culture | Survival | (Metcalf et al., 2023) |
| *Enterococcus faecalis* | Sea water and freshwater submerged with microplastic and glass particles | 14 days | 15°C (move sequentially from fresh water-estuary-marine-beach continuum) | Culture | Survival | (Metcalf et al., 2023) |
| *Enterococcus faecium* | Water sediment | 3 days | _ | Culture | Survival | (Young et al., 2019) |
| *Enterococcus* spp. | Fresh water sediment | 90 days | _ | Culture | Survival | (Haller et al., 2009) |
| *Enterococcus* spp. *(Entero 1 gene)* | Tidal creek | 3 months | 4.46-38.56°C | qPCR | Survival | (Jones et al., 2018) |
| *E. coli* | River water | 37 days | 8°C, 18°C | Culture | Survival | (Essert et al., 2023) |
| *E. coli O157:H7* | Untreated river water | 109 days | 37°C, pH 2.5 - 3.0 | Culture | Survival | (Scott et al., 2006) |
| *Fecal Coliforms & Enterococci* | Surface water (Terrieu Creek) and the groundwater | 4-5 days | 25°C (After rainfall) | Culture | Survival | (Mahler et al., 2000) |
| *Fecal indicators bacteria (FIB)* | Stream water | 25 days | 18°C | qPCR | Survival | (Haack et al., 2015) |
| *Francisella tularensis (subsp.halarctica & subsp. Tularensis)* | Saline solution | 24 weeks | 4°C | Culture | Survival | (Golovliov et al., 2021). |
| *Francisella tularensis (subsp.halarctica & subsp. Tularensis)* | Saline solution | 14 weeks | 20°C | Culture | Survival | (Golovliov et al., 2021). |
| *Helicobacter pylori* | Drinking water biofilm | 31 days | 15 and 20°C | Culture and qPCR | Survival | (Gião et al., 2008) |
| *Human associated bacteroidates* | Sewage water | 28 days | 25°C | qPCR | Survival | (McQuaig et al., 2009) |
| *Klebsiella oxytoca* | River water | 37 days | 8°C | Culture | Survival | (Essert et al., 2023) |
| *Klebsiella pneumoniae (hypermucoviscous)* | Fresh water microcosms | 44 days | 10°C & 20°C | Culture | Survival | (Soto et al., 2020) |
| *Klebsiella pneumoniae (nonhypermucoviscous)* | Fresh water microcosms | 14 days | 10°C & 20°C | Culture | Survival | (Soto et al., 2020) |
| *Klebsiella pneumoniae(hypermucoviscous and nonhypermucoviscous)* | Sea water | 7 days | 10°C & 20°C | Culture | Survival | (Soto et al., 2020) |
| *Legionella pneumophila* | Drinking water | 850 days | 22 ± 1°C (room temperature) | Culture and qPCR | Persistence | (Shaheen *et al.*, 2019) |
| *Leptospira interrogans serovar* | Spring water | 28 days | 29°C | Culture and qPCR | Survival | (Casanovas-Massana et al., 2018) |
| *Leptospira interrogans serovar* | Sewage water | 8 days | 29°C | Culture and qPCR | Survival | (Casanovas-Massana et al., 2018) |
| *Listeria innocua* | Stored irrigation water | 28 days | 3-11°C  pH 7.5 | Culture and qPCR | Survival | (Machado-Moreira et al., 2021) |
| *Listeria monocytogenes* | River water | 28 days | 5, 25 & 37°C | Culture | Survival | (Kabir et al., 2020) |
| *Listeria monocytogenes* | Drinking water & biofilm | 35 - 47 days | 10°C | Culture | Survival | (Bjergbæk et al., 2021) |
| *Listeria monocytogenes* | Seaweed associated water | 7 days | 4°C,10°C | Culture | Survival | (Akomea-Frempong et al., 2023) |
| *Listeria monocytogenes* | Seaweed associated water | 8 hours | 22°C | Culture | Survival | (Akomea-Frempong et al., 2023) |
| *Listeria monocytogenes* | Drinking water | At least 28 days | 10°C | Culture | Survival | (Bjergbæk et al., 2021) |
| *Methanobrevibacter smithii* | Sewage water | 28 days | 25 and 35°C | qPCR | Survival | (McQuaig et al., 2009) |
| *Mycobacteria avium(non tuberculosis mycobacteria)* | Drinking water | 26 months | 9.9-21.5°C | qPCR | Persistence | (Hilborn et al., 2006) |
| *Mycobacterium ulcerans DNA* | Water detritus | above 27 months | 8°C | qPCR | Persistence | (Bratschi et al., 2014) |
| *Mycoplasma bovis* | Shaded water | 6 hours | 15.17°C | qPCR | Survival | (Johnson et al., 2022) |
| *Mycoplasma bovis* | Water exposed to sunlight | 2 hours | 19.09°C | qPCR | Survival | (Johnson et al., 2022) |
| *Non HMV Klebsiella pneumoniae* | Fresh water microcosms | 14 days | 20°C | Culture | Survival | (Soto et al., 2020) |
| *Non HMV Klebsiella pneumoniae* | Marine water | 7 days | 20°C | Culture | Survival | (Soto et al., 2020) |
| *Non-typhoidal Salmonella enterica serovars* | River water | 28 days | 5, 25 & 37°C | Culture | Survival | (Kabir et al., 2020) |
| *Pseudomonas aeruginosa* | Sink drain water (Boiling water) | 2 -4 weeks | _ | Culture and qPCR | Survival | (Bourdin et al., 2024) |
| *Pseudomonas aeruginosa* | Sea water and freshwater submerged with microplastic and glass particles | >27 days | 15°C | Culture | Survival | (Metcalf et al., 2023) |
| *Pseudomonas aeruginosa* | Sea water and freshwater submerged with microplastic and glass particles | >14 days | 15°C(move sequentially from fresh water -estuary-marine-beach continuum) | Culture | Survival | (Metcalf *et al.*, 2023) |
| *Pseudomonas aeruginosa* | Atlantic Ocean | 1 month | 20°C | Culture | Survival | (Shteinberg et al., 2015) |
| *Pseudomonas aeruginosa* | Dead sea | 24 hours | 20°C | Culture | Survival | (Shteinberg et al., 2015) |
| *Salmonella* | Spring lake water | 1-7 days | - | qPCR | Survival | (Gaertner et al., 2011) |
| *Salmonella* | Spring lake water | 23 days | _ | qPCR | Survival | (Gaertner et al., 2011) |
| *Salmonella* | Reverse osmosis water matrix (lime stabilization) | 6 minutes | 28°C  pH 11.5-12.0 | Most probable number | Survival | (Bean et al., 2007) |
| *Salmonella enterica* | Sterile and nonsterile water | 28 days | 15°C | qPCR | Survival | (Banihashemi *et al.*, 2017) |
| *Salmonella enterica subsp. enterica serotypes* | Nuclease-free water | 160 days | 4°C, 25°C | Culture | Survival | (Williams et al., 2024). |
| *Salmonella entrica serovar typhimurium* | Filtered sea water | 74 weeks | 12°C | Culture | Persistence | (Davidson et al., 2015) |
| *Salmonella* sp. | Tidal creek | 3 months | 4.46-38.56°C | qPCR | Survival | (Jones et al., 2018) |
| *Salmonella* spp. | Seaweed associated water | 7 days | 4°C,10°C | Culture | Survival | (Akomea-Frempong et al., 2023) |
| *Salmonella* spp. | Seaweed associated water | 8 hours | 22°C | Culture | Survival | (Akomea-Frempong et al., 2023) |
| *Salmonella* spp. | Untreated irrigation water | 6 days | 20°C | Culture | Survival | (Van Der Linden et al., 2014) |
| *Salmonella* spp. | Untreated irrigation water | 14 days | 4°C | Culture | Survival | (Van Der Linden et al., 2014) |
| *Salmonella typhimurium* | Microcosm with stream water | >142 hours | 30°C (free floating & particle) | Culture | Survival | (Wang et al., 2018) |
| *Salmonella typhimurium* | Microcosm with stream water | >120 hours | 40°C (free floating) | Culture | Survival | (Wang et al., 2018) |
| *Salmonella typhimurium* | Microcosm with stream water | >164 hours | 40°C (sediment particle attached) | Culture | Survival | (Wang et al., 2018) |
| *Salmonella typhimurium* | Microcosm with stream water | 5 hours | 50°C (free floating) | Culture | Survival | (Wang et al., 2018) |
| *Salmonella typhimurium* | Microcosm with stream water | >21 hours | 50°C (sediment particle attached) | Culture | Survival | (Wang et al., 2018) |
| *Salmonella typhimurium* | Microcosm with stream water | 1.3 hours | 60°C (free floating) | Culture | Survival | (Wang et al., 2018) |
| *Salmonella typhimurium* | Microcosm with stream water | 2 hours | 60°C (sediment particle attached) | Culture | Survival | (Wang et al., 2018) |
| *Salmonella typhimurium* | Untreated irrigation water | 14 days | 4°C & 20°C | Culture | Survival | (Van Der Linden et al., 2014) |
| *Salmonella typhimurium* | Unfiltered sea water | 32 weeks | 12°C | Culture | Survival | (Davidson et al., 2015) |
| *Salmonella typhimurium* | Sea water microcosms | 12 months | pH 8 | Culture and qPCR | Persistence | (Chakroun et al., 2017) |
| *Serratia marcescens* | Sink drain water | 1-4 weeks | - | Culture and qPCR | Survival | (Bourdin et al., 2024) |
| *Shigatoxigenic E.coli (STEC)* | Seaweed associated water | 7 days | 4°C,10°C | Culture | Survival | (Akomea-Frempong et al., 2023) |
| *Shigatoxigenic E.coli (STEC)* | Seaweed associated water | 8 hours | 22°C | Culture | Survival | (Akomea-Frempong et al., 2023) |
| *Shigella* spp.*(ipaH)* | Tidal creek | 11 days | 4.46-38.56°C | qPCR | Survival | (Jones et al., 2018) |
| *Spores of sulfite-reducing clostridia* | River water | > 42 days | 20 ± 2°C  pH 7.4 - 8.0 | Culture | Survival | (Martín-Díaz et al., 2017) |
| *Staphylococcus aureus* | River water | 7 days | 8°C | Culture | Survival | (Essert et al., 2023) |
| *Staphylococcus aureus* | River water | 3 days | 18°C | Culture | Survival | (Essert et al., 2023) |
| *Stenotrophomonas maltophilia* | Sink drain water | 3 weeks | - | Culture and qPCR | Survival | (Bourdin et al., 2024) |
| *Vancomycin-resistant enterococci (VRE) DNA* | Water sediment | 12 days | _ | Culture | Survival | (Young, Rohr and Harwood, 2019) |
| *Vancomycin-resistant enterococci faecium strain* | Filtered river water | 7 days | _ | Culture | Survival | (Young, Rohr and Harwood, 2019) |
| *Vibrio parahaemolyticus* | Artificial seawater | 56 days | 18°C | Culture | Survival | (Vasudevan and Venkitanarayanan, 2006) |
| *Vibrio parahaemolyticus* | Artificial seawater | 21 days | 6°C | Culture | Survival | (Vasudevan and Venkitanarayanan, 2006) |
| *Vibrio parahaemolyticus* | Artificial seawater | 12 days | 4°C,8°C | Culture | Survival | (Vasudevan and Venkitanarayanan, 2006) |
| *Vibrio parahaemolyticus strain* | Sea water | 72 hours | 22°C | Culture | Survival | (Richards et al., 2012) |
| Vibrio spp. | Seaweed-associated water | 7 days | 4°C, 10°C | Culture | Survival | (Akomea-Frempong et al., 2023) |
| Vibrio vulnificus | Natural Seawater | 72 hours | - | Culture | Survival | (Richards et al., 2012) |
| *Yersinia enterocolitica* | Sterile and nonsterile river water | 28 days | 15°C | qPCR | Survival | (Banihashemi *et al.,* 2017) |

**Table S2**. Survival and persistence of viruses in various water types/sources

| **Virus** | **Water Source** | **Duration** | **Condition** | **Method of Detection** | **Persistence/Survival** | **Reference** |
| --- | --- | --- | --- | --- | --- | --- |
| Avian influenza virus (LPAIV) HINI | Artificial stream | 14 days | 10-13°C, 16-18°C | Culture and qPCR | Survival | (Perlas et al., 2023) |
| Human Adenovirus | Sewage water | 28 days | 25 and 35°C | qPCR | Survival | (McQuaig et al., 2009) |
| Human Adenovirus | Ground water | > 3years | - | - | Persistence | (Anderson et al., 2011) |
| Human Adenovirus | Surface water | >70 days | - | - | Survival | (Anderson et al., 2011) |
| Human adenovirus (40/41) | Unfiltered Lake water microcosm | 28 days | 15°C,25°C | qPCR | Survival | (Ahmed et al., 2021) |
| Human Adenovirus type 5 | Reverse osmosis water matrix (lime stabilisation) | 6 minutes | 28°C,  pH 11.5-12.0 | Tissue culture infectious dose | Survival | (Bean et al., 2007) |
| Human Norovirus | Surface water | >70 days | 37°C | qPCR | Survival | (Anderson et al., 2011) |
| Human Norovirus | Ground water | >3years | - | qPCR | Persistence | (Anderson et al., 2011) |
| Human Polyomaviruses | Sewage water | 28 days | 25 and 35°C | qPCR | Survival | (McQuaig et al., 2009) |
| Murine Norovirus | Sterile demineralised water | >42 days | 4°C | Culture & qPCR | Survival | (Zhu et al., 2020) |
| Murine Norovirus | Phosphate‐buffered saline aqueous water | >14 days | 25°C (interaction with Acanthamoeba spp.) | Culture | Survival | (Hsueh and Gibson, 2015) |
| Vaccinal Poliovirus Type 1 (infectious) | Drinking water biofilm | 6 days | 10°C, pH 7.67 | Buffalo green monkey (BGM) | Survival | (Helmi et al., 2008) |
| Porcine adenovirus (Padv) | Stream water | 18 days | _ | qPCR | Survival | (Haack *et al.*, 2015) |
| Porcine epidemic diarrhea virus | Sea water | 28 days | 4-8°C | Immunoperoxidase monolayer assay | Survival | (Contrant et al., 2023) |
| Porcine epidemic diarrhea virus | Sea water | 28 days | 15°C | Immunoperoxidase monolayer assay | Survival | (Contrant et al., 2023) |
| Porcine epidemic diarrhea virus | Sea water | 7 days | 25°C | Immunoperoxidase monolayer assay | Survival | (Contrant et al., 2023) |
| Porcine teschovirus (PTV) | Stream water | 18 days | _ | qPCR | Survival | (Haack *et al.*, 2015) |
| Rotavirus | Reverse osmosis water matrix (lime stabilization) | 6 minutes | 28 °C  pH 11.5-12.0 | Modified plaque-forming unit | Survival | (Bean et al., 2007) |
| SARS-COV | Wastewater & dechlorinated tap water | 2 days | 20°C | qPCR | Survival | (Basavaraju et al., 2021) |
| SARS-COV | Wastewater & dechlorinated tap water | 14 days | 4°C | qPCR | Survival | (Basavaraju et al., 2021) |
| Virulent Aeromonas hydrophila (vAh) | Pond sediment | 28 days | 28°C | Culture and qPCR | Survival | (Tuttle et al., 2023) |

**Table S3**. Survival and persistence of parasites and protozoans in various water types/sources

| **Parasite** | **Water Source** | **Duration** | **Condition** | **Method of Detection** | **Persistence/Survival** | **Reference** |
| --- | --- | --- | --- | --- | --- | --- |
| *Ascaris lumbricoides ova* | Reverse osmosis water matrix (lime stabilization) | 72 hours | 28°C  pH 11.5-12.0 | Observation of motile larvae under 40x magnification | Survival | (Bean et al., 2007) |
| *Colpoda* spp. | Chlorinated water and Underground water | 6 months | 3°C-21°C | Denaturing gradient gel electrophoresis | Survival | (Baré et al., 2011) |
| *Cryptosporidium parvum* | Drinking water biofilm | 34 days | 10°C, pH 7.67 | Epifluorescence microscopic observation using a fluorescein-labelled double monoclonal antibody | Survival | (Helmi et al., 2008) |
| *Cryptosporidium parvum* | Drinking water without biofilm | 7 days | 10°C, pH 7.67 | Epifluorescence microscopic observation using a fluorescein-labelled double monoclonal antibody | Survival | (Helmi et al., 2008) |
| *Cryptosporidium parvum* (gene) | Filtered & Unfiltered water | 28 days | 15°C,25°C | qPCR | Survival | (Ahmed et al., 2021) |
| *Cryptosporidium parvum* (oocyst) | Filtered & Unfiltered water | 28 days | 15°C,25°C | Vital dye staining | Survival | (Ahmed et al., 2021) |
| *Cryptosporidium parvum* (oocyst) | Reverse osmosis water matrix (lime stabilization) | 72 hours | 28°C  pH 11.5-12.0 | Immunofluorescent antibody staining | Survival | (Bean et al., 2007) |
| *Giardia lamblia* | Reverse osmosis water matrix (lime stabilization) | 48 hours | 28°C  pH 11.5-12.0 | Immunofluorescence | Survival | (Bean et al., 2007) |
| *Giardia lamblia* | Drinking water biofilm | >34 days | _ | Epifluorescence microscopic observation using a fluorescein-labeled double monoclonal antibody | Survival | (Helmi et al., 2008) |
| *Naegleria* spp. | Drinking water (in poultry house) | 6 months | 3°C-21°C | qPCR | Survival | (Baré et al., 2011) |
| *Trichobilharzia szidati cercariae* | Dechlorinated tap water | 204 hours | 5°C | Morphometric characters & PCR | Survival | (Al-Jubury et al., 2020) |
| *Trichobilharzia szidati cercariae* | Dechlorinated tap water | 108 hours | 10°C | Morphometric characters & PCR | Survival | (Al-Jubury et al., 2020) |
| *Trichobilharzia szidati cercariae* | Dechlorinated tap water | 96 hours | 15°C | Morphometric characters & PCR | Survival | (Al-Jubury et al., 2020) |
| *Trichobilharzia szidati cercariae* | Dechlorinated tap water | 60 hours | 20°C | Morphometric characters & PCR | Survival | (Al-Jubury et al., 2020) |
| *Trichobilharzia szidati cercariae* | Dechlorinated tap water | 48 hours | 25°C | Morphometric characters & PCR | Survival | (Al-Jubury et al., 2020) |
| *Trichobilharzia szidati cercariae* | Dechlorinated tap water | 36 hours | 30°C | Morphometric characters & PCR | Survival | (Al-Jubury et al., 2020) |
